# Rapid and efficient CRISPR/Cas9 gene inactivation in human neurons during human pluripotent stem cell differentiation and direct reprogramming

**DOI:** 10.1038/srep37540

**Published:** 2016-11-18

**Authors:** Alicia Rubio, Mirko Luoni, Serena G. Giannelli, Isabella Radice, Angelo Iannielli, Cinzia Cancellieri, Claudia Di Berardino, Giulia Regalia, Giovanna Lazzari, Andrea Menegon, Stefano Taverna, Vania Broccoli

**Affiliations:** 1 Division of Neuroscience, San Raffaele Scientific Institute, 20132 Milan, Italy; 2 Advanced Light and Electron Microscopy Bio-Imaging Centre, Experimental Imaging Centre, San Raffaele Scientific Institute, 20132 Milan, Italy; 3 Neuroengineering and medical robotics laboratory, Department of Electronics, Information and Bioengineering, Politecnico di Milano, 20133 Milan, Italy; 4 Avantea srl, Laboratory of Reproductive Technologies, Via Porcellasco 7f, 26100 Cremona, Fondazione Avantea, Cremona; 5 Neuroimmunology Unit, San Raffaele Scientific Institute, 20132 Milan, Italy; 6 National Research Council (CNR), Institute of Neuroscience, Milan, Italy

## Abstract

The CRISPR/Cas9 system is a rapid and customizable tool for gene editing in mammalian cells. In particular, this approach has widely opened new opportunities for genetic studies in neurological disease. Human neurons can be differentiated *in vitro* from hPSC (human Pluripotent Stem Cells), hNPCs (human Neural Precursor Cells) or even directly reprogrammed from fibroblasts. Here, we described a new platform which enables, rapid and efficient CRISPR/Cas9-mediated genome targeting simultaneously with three different paradigms for *in vitro* generation of neurons. This system was employed to inactivate two genes associated with neurological disorder (*TSC2* and *KCNQ2*) and achieved up to 85% efficiency of gene targeting in the differentiated cells. In particular, we devised a protocol that, combining the expression of the CRISPR components with neurogenic factors, generated functional human neurons highly enriched for the desired genome modification in only 5 weeks. This new approach is easy, fast and that does not require the generation of stable isogenic clones, practice that is time consuming and for some genes not feasible.

Human pluripotent stem cells (hPSCs, including both embryonic stem (ES) and induced pluripotent stem cells (iPSCs)) are an invaluable *in vitro* platform for modeling neuronal diseases given the success in developing protocols for their differentiation into different neuronal cell types^1^,^2^,^3^. In fact, functional studies can be applied to iPSC-derived neurons reprogrammed from patient somatic cells in order to decipher the pathological mechanisms in the proper affected neuronal cell^4^,^5^,^6^. The CRISPR/Cas9 (clustered, regularly interspaced, short palindromic repeats (CRISPR)/CRISPR associated (Cas)) system has rapidly become the most preferred genome-editing tool given its highly precise and efficient targeting, easy experimental design and straightforward implementation^7^,^8^,^9^,^10^,^11^. The identification of new Cas9 orthologs and their engineered variants with different size and PAM (protospacer adjacent motif) specificity has significantly extended the flexibility of the system and its targeting range across the genome^11^,^12^,^13^,^14^. This nuclease system is particular advantageous for disease modeling since it permits the generation of isogenic hPSC clones that differ only in the gene of interest bypassing the intrinsic variability of iPSC lines derived from different patients^15^,^16^,^17^. However, some hurdles in this experimental approach have to be taken into account. The generation of edited neurons is a long, expensive and time-consuming process. hPSCs have to be genetically modified, isolated and expanded to generate homogeneous clones before identifying if the mutation is present^18^. The selected clones have to be, then, differentiated into neurons to undertake the analysis of the pathophysiological defects related to the genetic mutation. This process can be even more cumbersome when the efficiency of the genetic modification of interest is particularly low or when multiple genes are targeted. Moreover, CRISPR/Cas9 technology can be hardly applied to cells more differentiated than hPSCs since their reduced self-renewal ability prevent the generation of clones starting from single cells. This approach is also unfeasible in cases where the inactivated gene could alter hPSC proliferation, pluripotency or differentiation capabilities. In addition, introducing a genetic modification in cells derived from hPSCs, such as neuronal precursor cells (hNPCs), can be beneficial to avoid the known difficulties of maintaining hPSCs in culture and their variability with passages^19^. To address some of these limitations, we conceived a fast and efficient approach to obtain human mutated neurons. This protocol is based on the introduction of targeted genome modifications using the CRISPR/Cas9 technology coupled to an accelerated neuronal differentiation protocol. Importantly, our method can be applied to hPSCs or hNPCs, and, with small adjustments, it can be also useful in other differentiation paradigms such as the direct conversion of somatic cells into post-mitotic neurons. Our group and others have developed protocols to obtain specific subtypes of neurons from fibroblast direct reprogramming without passing through an induced pluripotent stem cell^20^,^21^,^22^,^23^,^24^,^25^,^26^. Direct reprogramming can represent an interesting alternative strategy for neuronal modeling^27^,^28^ in particular for late-onset neurological diseases since hPSCs generate immature neurons that may need long time in culture to recapitulate the disease phenotype^29^,^30^.

Herein, we sought to inactivate the *TSC2* and the *KCNQ2* genes whose mutations can cause severe neuropathologies in humans. These genes were selected based on the following criteria: (i) their mutations are associated to a loss-of-function disorder that affects predominantly neurons, (ii) they are responsible for a genetic dominant disease, (iii) the disease presents well-established defects that could be studied *in vitro* and (*iv*) in mice models bi-allelic inactivation recapitulates these defects as much as mono-allelic mutation^31^,^32^,^33^. Mutations in *TSC2* are responsible for Tuberous Sclerosis, a disorder characterized by intellectual disability and seizures. Most patients have mutations in either the *TSC1* or *TSC2* gene but *TSC2*-dependent disease is more severe^33^. Inactivation of *TSC2*, or its homolog *TSC1* with whom it forms a multimeric complex, causes hyperactivation of the mTOR complex 1 (mTORC1) and hyperphosphorylation of its downstream effectors including the ribosomal S6 protein^34^,^35^,^36^. *KCNQ2* codifies for a voltage-gated potassium channel that mediates the M-current together with other *KCNQ* family members. M-currents ensure that the neuron is not constantly active and excitable^37^. Consistent with this role, mutations in *KCNQ2* and *KCNQ3* are associated with mild to severe early-onset epilepsy^38^, with deletions in *KCNQ2* being more detrimental for the formation of M-current than *KCNQ3*^39^. In this work, we demonstrate that the resulting mutated neurons present functional defects indicating the value of our methodological approach to analyze cellular and molecular phenotypes associated with neuronal disorders using a manageable procedure and very short timeframe.

## Results

### Generation of *KCNQ2* targeted human neurons and functional assessment

For an efficient inactivation of the *TSC2* and *KCNQ2* genes, we reasoned that the best sequence to target for the CRISPR/Cas9 gene inactivation was a region codifying for an essential functional domain of the protein, preferably enriched for disease-causing mutations, since a frameshift in this precise region would likely generate a complete loss-of-function. In addition, this particular design would reduce the generation of functional protein also in presence of in-frame mutations. For *KCNQ2* gene mutagenesis, we selected a sequence within exon 6, coding for the essential P-loop domain of the transmembrane channel pore since many disease-causing mutations are clustered in this domain^37^. More precisely, with on-line tool http://crispr.mit.edu we searched the sgRNAs present in the sequence around the Y284C mutation and selected only high quality guides (score ≥ 50%, inversely proportional to off-target predicted activity)^9^. Among them we designed 6 different sgRNAs (Figure S1a, Supplementary Table 1). Targeting efficiency was then taken into consideration as further criterion to select them. To determine the cleavage efficiency of the chosen sgRNAs, we first engineered an LV-U6 lentiviral vector containing each single sgRNA and infected a SpCas9-expressing HEK293T stable cell line previously established (Figure S1b). Two days after infection, DNA cleavage efficiency was assessed using the T7 Endonuclease I (T7EI) assay. As shown in Figure S1c, the sgRNA-K6 triggered the highest cleavage rate (almost 70%) and was chosen for the following functional experiments (Figure S1d).

**Figure 1:**
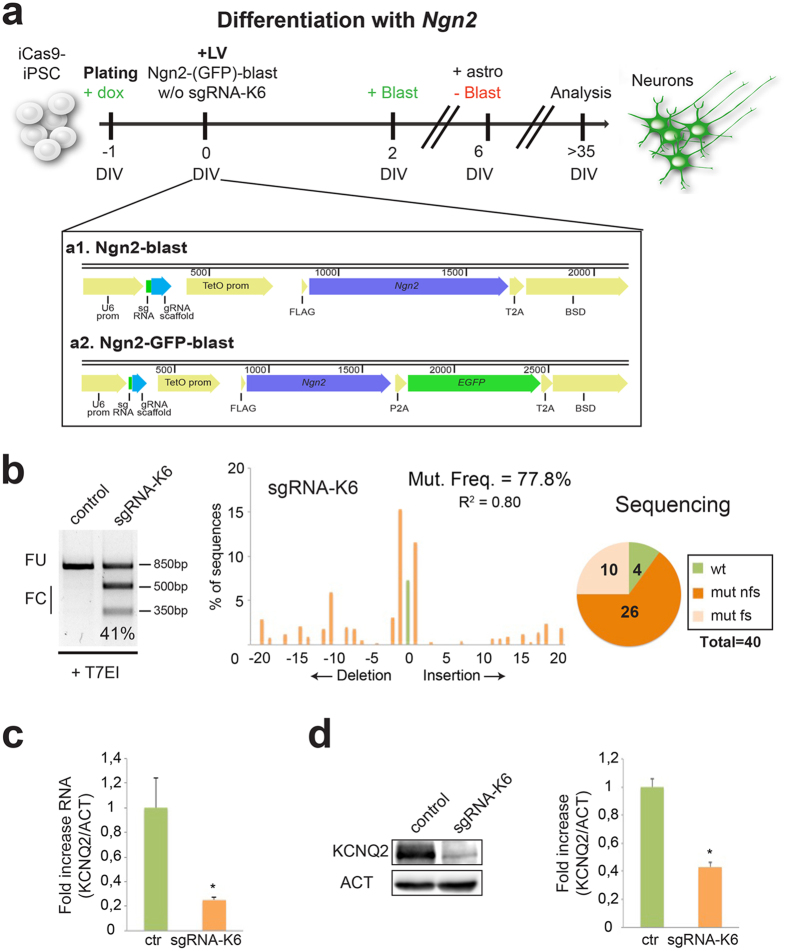
*KCNQ2* gene inactivation by CRISPR/Cas9 strongly reduces the expression of KCNQ2 in hPSCs-derived neurons. (**a**) General strategy to generate an enriched population of *KCNQ2*-targeted neurons from iCas9- hPSCs. The reprogramming protocol, based on the forced expression of *Ngn2*, is coupled to the mutagenesis of the *KCNQ2* gene when the sgRNA-K6 guide is transduced. Doxycycline was added into the media at DIV-1 and it was maintained for all the experiment. Blasticidin was added to select the transduced cells (from DIV2 to DIV6). Bottom **a**1: schematic illustration of the Ngn2-blast lentiviral vector expressing sgRNA, *Ngn2* and blasticidin resistance gene (BSD) linked by a T2A sequence. Bottom **a**2: schematic illustration of the Ngn2-GFP-blast lentivirus expressing sgRNA, *Ngn2*, *EGFP* and BSD linked by P2A and T2A domains. (**b**) T7EI analysis in control and sgRNA-K6 neurons. FC and FU indicate the expected fraction cleaved and uncleaved used to quantify the indels, respectively. Middle, TIDE assay of the sgRNA-K6 neurons. In all figures R^2^ indicates the variance, a statistic value of likelihood of the TIDE prediction. Right, number of wild type (wt), frameshift mutations (mut fs) and non frameshift mutations (mut nfs) found among 40 sequences analyzed in sgRNA-K6 neurons. (**c**) Quantitative RT-PCR for the expression of *KCNQ2* in control and sgRNA-K6 neurons. Data were normalized to the reference gene *β-ACTIN* (ACT). In all figures, data are presented as means ± sem (in this case, n = 3, *p < 0.05). (**d**) Western-blots to detect KCNQ2 and ACTIN protein levels in control and sgRNA-K6 neurons. Densitometric quantification of KCNQ2 relative to ACTIN protein levels in arbitrary units (n = 3, *p < 0.05). Full-length gel and blots are included in Figure S7a,b.

To develop a rapid and efficient CRISPR/Cas9 system to target genes implicated in neuronal pathologies, we thought that coupling the neuronal differentiation to gene inactivation would represent a very convenient system to model human diseases. To reach this dual aim, we employed hPSCs targeted with a doxycycline (dox)-inducible SpCas9 gene^40^ and combined with an accelerated neuronal differentiation method based on the forced expression of *Ngn2*^41^. Thus, we cloned a multicistronic cassette to co-express *Ngn2* and the blasticidin resistance gene (Ngn2-blast) in lentiviral vector with or without the sgRNA-K6 (Fig. 1a). iCas9-hPSCs were dissociated in single cells and seeded in medium containing dox to induce SpCas9 expression and, after 24 hours, we transduced the hPSCs with the Ngn2-blast lentiviral particles expressing or not (control) the sgRNA-K6 (Fig. 1a). Transduced iCas9-hPSCs were selected for blasticidin resistance for 5 days and then differentiated in a neurobasal medium supplemented with B27 and BDNF. 5 weeks after the infection, the indel frequency was assessed in the neuronal population. Ngn2-blast-sgRNA-K6 transduced iCas9-hPSC-derived neurons showed a 40% indel frequency based on T7EI assay which raises to about 75% for the TIDE prediction (38. 7 ± 1.5% for T7EI vs. 71.7 ± 4.9% for TIDE, n = 3; Fig. 1b). However, 90% of the individual DNA molecules from the targeted genomic region resulted mutated by direct sequencing with 72% harboring out-of-frame indels (Figs 1b and S2). These findings confirmed that the T7EI assay can strongly underestimate the indel frequency as suggested in previous studies^40^. To evaluate the *KCNQ2* gene targeting specificity, we sequenced the 8 most likely off-target sites within the genes with less than 5 mismatches and, thereby, the highest probability of binding to the sgRNA-K6 as predicted by the CRISPR Design Tool (Figure S1c). Both TIDE analysis on the sequencing traces from the bulk population and direct sequencing of cloned amplicons failed to identify any mutation in the 8 genes (Figure S1c and data not shown). Thus, the Cas9/sgRNA-K6 pair resulted highly efficient in targeting the *KCNQ2* gene with considerable sequence specificity.

To study the expression of *KCNQ2* in the mutated neurons, we performed a quantitative RT-PCR and a western blot. Both techniques indicated that KCNQ2 expression was reduced in the sgRNA-K6 neurons respect to the controls (Fig. 1c,d).

**Figure 2:**
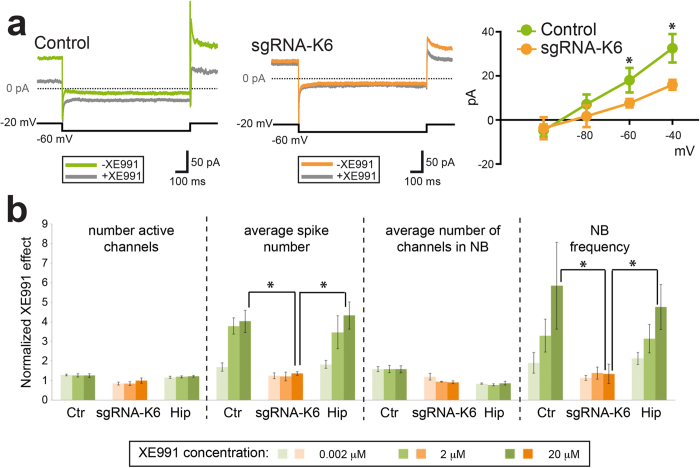
*KCNQ2* gene inactivation by CRISPR/Cas9 impaires M-currents in iCas9- hPSCs-derived neurons. (**a**) Voltage-clamp recordings of XE991-sensitive currents. Left, examples of traces recorded from a control neuron in response to a 1-s voltage step from −20 mV to −60 mV before and after extracellular perfusion with 20 μM XE991. Middle, traces obtained in the same conditions in a sgRNA-K6-targeted neuron. Right, current-voltage plot of steady-state amplitudes of XE991-sensitive currents in control and sgRNA-K6-treated neurons (n = 10 and 7, respectively, *p < 0.05). (**b**) Pharmacological study of the activity of the KCNQ2 channel in hPSC-derived neurons (ctr and sgRNA-K6) and primary hippocampal neurons (hip) by extracellular electrophysiology. XE991 has been used in three different concentrations (0.002, 2, 20 μM) represented by the increasing intensity of colours. Four functional parameters are shown: the number of active channels, the average spike number, the average number of channels involved in a network burst and the network burst frequency. Each bar represents the average value ± SEM on a 10-minute window of recording for each parameter and XE991 concentration (n = 3 for ctr and sgRNA-K6, n = 8 for hip, *p < 0.05).

To test the functional defects of the mutated bulk neuronal population, we performed whole-cell patch-clamp recordings to test M-current properties in sgRNA-K6 and control treated hPSC-derived neurons. For these analyses, murine astrocytes were added to the cultures to promote neuronal functional maturation and iCas9-hPSCs were infected with a modified version of the Ngn2-blast viral construct containing the EGFP coding gene (Ngn2-GFP-blast) to facilitate their identification by fluorescence visualization. The current was pharmacologically isolated by adding ACSF with XE991, a potent and selective KCNQ2/3 channel blocker which causes neuronal hyperexcitability in a dose-sensitive manner (see Methods). In voltage-clamp conditions, current responses to four negative voltage steps from a holding potential of −20 mV to −100 mV (in 10-mV steps) were recorded before and after extracellular perfusion with 20 μM XE991 (Fig. 2a, left and middle panels). Average steady-state amplitudes of the XE991-sensitive M-current recorded at −40 and −60 mV were significantly larger in control with respect to sgRNA-K6 treated hPSC-derived neurons (−40 mV: 32 ± 6 pA *vs*. 16 ± 2 pA, respectively; −60 mV: 18 ± 6 pA *vs*. 8 ± 2 pA, respectively, p < 0.05, unpaired t test, Fig. 2a). Next, we asked whether the impaired response to XE991 could be evaluated on spontaneous electric activity in the whole neuronal network. We, thus, co-cultured sgRNA-K6 and control treated human neurons together with primary mouse astrocytes on microelectrode arrays (MEAs) to enable dynamic recordings over time. We monitored the activity of the whole neuronal cultures by quantifying the number of active channels, the average spike number, the average number of channels involved in network bursts (NB) and the NB frequency^42^. Increasing doses of XE991 (0.002, 2, 20 μM) in both mouse primary hippocampal and hPSC-derived neuronal cultures did not affect the parameters describing the structure of the neuronal network (n° of active channels, n° of channels in NB), while elicited a rapid increase in firing activity (spike number) and its synchronization (NB frequency) (Fig. 2b). In contrast, sgRNA-K6 treated hPSC-derived neurons resulted not responsive at any XE991 concentration (Fig. 2b). In particular, at the highest dose of XE991 (20 μM), comparable high levels of hyperexcitability were recorded in control hPSC-derived and mouse primary neurons, but not in sgRNA-K6-treated hPSC- derived neurons (p < 0.05) (Fig. 2b). These observations demonstrated that CRISPR-mediated *KCNQ2* gene silencing treatment was effective in silencing M-currents in human neurons leading to a predictable lack of functional response to its channel blocker as assessed at both single cell and neuronal network level.

### CRISPR-mediated *TSC2* gene mutagenesis in hPSC-derived neurons

For efficient *TSC2* gene inactivation, we decided to target exon 37 since it encodes for the GAP domain of the TSC2 protein and is associated to numerous mutations causing Tuberous Sclerosis^43^. We selected 6 different sgRNAs overlapping or close to the Y1571X mutation^43^ based on their low off-target activity (Supplementary Table 1), and then evaluated their targeting efficiency in Cas9-expressing HEK293T cells (Figure S3a). In T7EI analysis the sgRNA-T4 was the guide triggering the highest DNA cleavage efficiency (almost 65%) and was selected for the subsequent studies (Figure S3b,c).

**Figure 3:**
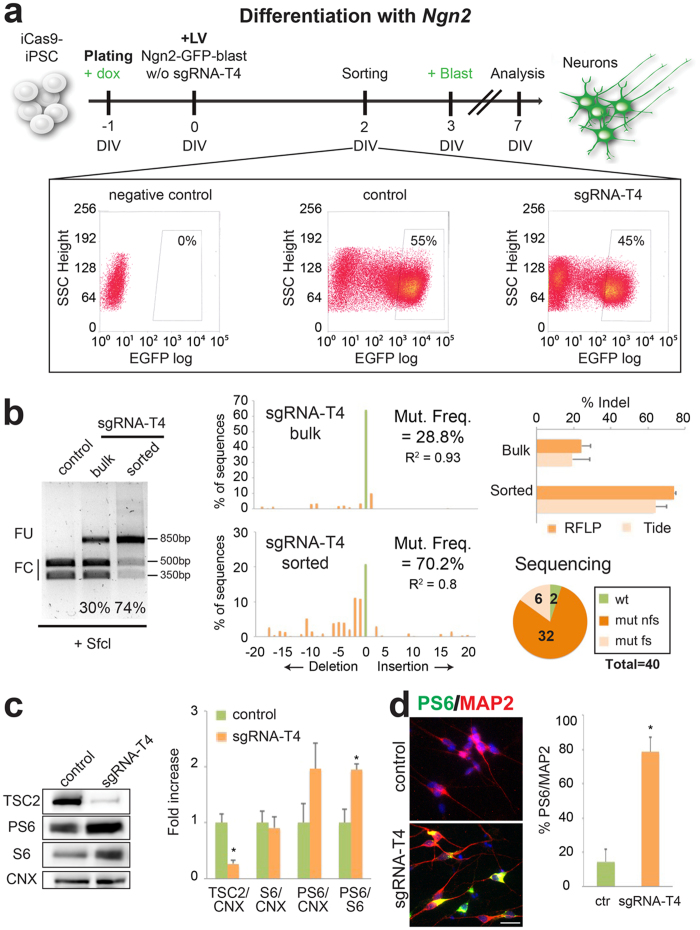
Rapid and efficient *TSC2* gene inactivation by CRISPR/Cas9 gene inactivation in iCas9- hPSC-derived neurons. (**a**) Strategy to obtain an enriched population of *TSC2*-edited neurons from iCas9-hPSCs. The differentiation protocol, based on the forced expression of *Ngn2*, is coupled to *TSC2* gene mutagenesis when the sgRNA-T4 guide is also transduced. Doxycycline (added at DIV-1) and blasticidin (DIV 3) were maintained for all the experiment. Representative FACS plots of EGFP levels in iCas9-hPSCs. The lentiviruses used to infect each sample were: Ngn2-blast for the negative control, Ngn2-GFP-blast for the control and Ngn2-GFP-blast-sgRNA-T4 for the sgRNA-T4 sample. The EGFP population selected is enclosed in the box. (**b**) RFLP assay performed in control sorted, sgRNA-T4 bulk and sgRNA-T4 sorted populations using the SfcI enzyme. Middle, representative TIDE assay for either the sgRNA-T4 bulk or sgRNA-T4 sorted populations. Right, indel quantification by RFLP and TIDE in sgRNA-T4 bulk and sorted cells (n = 2). Number of wild type (wt), frameshift mutations (mut fs) and non frameshift mutations (mut nfs) found among 40 sequences analyzed in sgRNA-T4 sorted cells. (**c**) Western-blots to detect TSC2, PS6, S6 and Calnexin (CNX) protein levels in control and sgRNA-T4 neurons. Densitometric quantification of TSC2, PS6 and S6 relative to CNX or S6 protein levels in arbitrary units (n = 4 for TSC2, n = 5 for the others, *p < 0.05). (**d**) Immunostaining of the sorted cells for MAP2 and PS6. Quantification of PS6/MAP2 double positive cells respect to the total MAP2-positive cell population (n = 3, *p < 0.05). Nuclei were stained with Hoechst. Scale bar: 20 μm. Full-length gel and blots are included in Figure S7c and d.

Next, we exploited the feasibility of our method to introduce targeted mutations during iCas9-hPSC accelerated neuronal differentiation forced by lentiviral *Ngn2* gene transduction.Initially, we designed and performed an RFLP assay using the SfcI enzyme to determine the loss of a restriction site close to the targeted region. RFLP analysis showed a quite limited indel rate (33.4 ± 4.4%, n = 5) on the derived differentiated neuronal population. To improve the system, we transduced iCas9-hPSCs with Ngn2-GFP-blast and then EGFP-positive cells were purified for EGFP expression by FACS-based cell sorting (≈50% of the total cell population, Fig. 3a), differentiated into neurons and examined for *TSC2* gene targeting. With this improvement, purified iCas9-hPSC-derived neurons exhibited a significant indel rate by RFLP and TIDE analyses (≈70%; Fig. 3b). These experiments indicate that the EGFP cell selection can strongly enriched the targeted cell population even when the selected sgRNA is not particular efficient in sustaining gene targeting. By sequencing the cloned DNA fragments from the bulk neuronal population, we found that 95% of the sequences were mutated, with 84% harboring out-of-frame indels (Figs 3b and S4). Multiple assays were necessary to detect cleavage efficiency, since they showed a different degree of accuracy (closeness to true value) or reproducibility (repeatability of the measurement). Indeed direct sequencing was the most accurate, but its repeatability often was not feasible. On the contrary T7EI assay was the least accurate but very reproducible, rapid and inexpensive, thus helpful when comparing different conditions (see sgRNA selection). The same can be said of RFLP analysis, but it had superior accuracy, although not matching direct sequencing (interestingly the presence of sgRNA-T4-mutated sequences that retained the SfcI site can explain this discrepancy, Figure S4, highlighted sequences), TIDE analysis showed accuracy and repeatability similar to RFLP analysis. We used all these methods to complement and validate each other in order to reach the most reliable description of the genetic modifications introduced.

Noteworthy, no alterations were detected in the sequences of the 8 most likely off-target sites localized on genomic sequences with less than 5 mismatches respect to the sgRNA-T4 target sequence (Figure S3d). Both TIDE analysis on the sequencing traces from the bulk population and direct sequencing of cloned amplicons failed to identify any mutation in the 8 genes (Figure S3d and data not shown). Thus, the Cas9/sgRNA-T4 pair resulted highly efficient in targeting the *TSC2* gene with considerable sequence specificity.

We, then, examined functional consequences in the *TSC2*-targeted human neurons. Since immunoblotting analysis required a substantial amount of cell lysates we used only material from the unsorted Ngn2-blast neuronal population. Despite the lower indel rate, we observed an evident reduction of total TSC2 protein (Fig. 3c). Loss of either *TSC1* or *TSC2* gene function abolishes Rheb-GTPase activity, resulting in constitutively activated mTORC1 kinase and consequently increased levels of the S6 ribosomal protein phosphorylated form (PS6)^34^. Remarkably, we observed a specific and robust increase in PS6 levels by immunoblotting (Fig. 3c). Accordingly, more PS6/MAP2 double positive cells were scored in the sgRNA-T4 compared to control targeted cells (Fig. 3d).

Next, we corroborated the validity of this method by assessing *TSC2* gene editing in mature neuronal cells differentiated from iCas9-hPSCs. After 7 days with *Ngn2*, neurons were infected with the sgRNA-T4 and analyzed by RFLP. In this setting the generation of indel mutations in post-mitotic human neurons was strongly inhibited most likely because in these cells transduction efficiency resulted very low (Figure S5a). In fact, EGFP was observed only in about 1% of the transduced neurons (Figure S5b). Thus, hPSCs, but not the differentiated neurons, constitute the ideal cellular platform for the CRISPR/Cas9-mediated gene targeting. This evidence raised a question about the kinetic of gene inactivation during our procedure. In order to evaluate it we performed a time-course analysis of sgRNA-T4 cleavage at DIV1, 2, 4, 6 and 8 of our protocol (Figure S5c). Indel rate reached the maximum level at DIV2 and then reached plateau, indicating that the occurrence of gene inactivation precede neuronal specification. We also questioned whether leakage from the TetO inducible system can be at least in part responsible for the targeting efficiency observed. No indel was detectable in absence of doxycycline (Figure S5d), thus ruling out this hypothesis.

**Figure 4:**
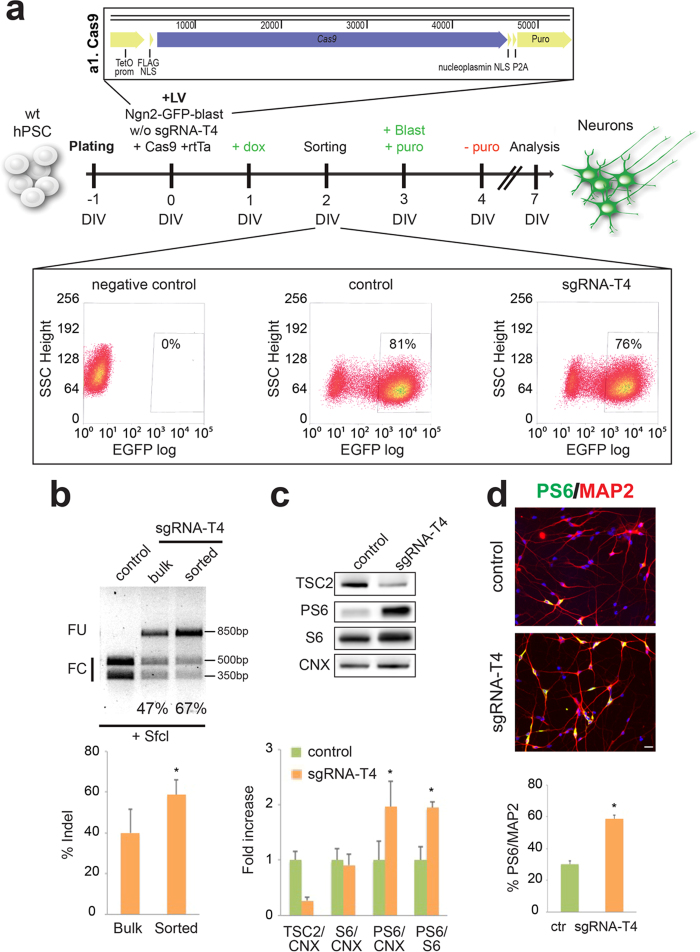
CRISPR/Cas9-mediated *TSC2* gene inactivation in wild-type hPSCs. (**a**) Top a1, illustration of the Cas9 lentiviral vector expressing Cas9 and puromycin resistance gene (Puro) under the TetO inducible promoter. Middle, schematic representation of the procedure to generate *TSC2* mutant neurons starting from wild-type hPSCs. Forced expression of *Ngn2* is coupled to *TSC2* gene inactivation by co-expressing the sgRNA-T4 guide and Cas9. Doxycycline (added at DIV1) and blasticidin (DIV 3) were maintained for all the experiment. Puromycin was added to select the transduced cells (from DIV3 to DIV4). Bottom, representative FACS plots of EGFP levels in wild-type hPSCs are presented. The lentiviruses used to infect each sample were: Ngn2-blast for the negative control, Ngn2-GFP-blast for the control and Ngn2-GFP-blast-sgRNA-T4 for the sgRNA-T4 sample. The EGFP positive population selected for the study is enclosed in the box area. (**b**) Representative SfcI RFLP assay performed in control sorted, sgRNA-T4 bulk and sgRNA-T4 sorted populations derived from wild-type hPSCs. Quantification of indel percentage by RFLP in sgRNA-T4 bulk and sgRNA-T4 sorted cells (n = 4, *p < 0.05). (**c**) Western-blots to detect TSC2, PS6, S6 and Calnexin (CNX) protein levels in control and sgRNA-T4 neurons. Densitometric quantification of TSC2, PS6 and S6 relative to CNX or S6 protein levels in arbitrary units (n = 3, *p < 0.05). (**d**) Immunostaining of the sorted cells for MAP2 and PS6. Quantification of PS6/MAP2 double positive cells respect to the total MAP2-positive cell population (n = 3, *p < 0.05). Nuclei were stained with Hoechst. Scale bar: 20 μm. Full-length gel and blots are included in Figure S7e and f.

Finally, we adapted this method in order to efficiently introduce mutations in the *TSC2* gene during neuronal differentiation of wild-type hPSCs. We transduced these cells with the lentivirus Ngn2-GFP-blast (with or without the sgRNA-T4), rtTA and another lentivirus co-expressing the SpCas9 gene coupled to a doxycycline-inducible promoter and the puromycin resistance gene to select the transgene-expressing cells (Fig. 4a). Then EGFP-positive cells were differentiated into neurons and examined for *TSC2* gene targeting by RFLP assay, obtaining an indel rate of about 60% (Fig. 4b). In the mutated cellular population we observed by western blot a reduction in TSC2 levels and an increase in PS6 (Fig. 4c). We also found increased numbers of PS6 neurons in sgRNA-T4 samples (Fig. 4d). This result confirmed the possibility to apply this system for evaluating the effects of gene inactivation in human neurons derived from not only iCas9-hiPSCs but also unmodified hPSCs.

### Combining hNPCs neuronal differentiation with CRISPR-mediated *TSC2* gene inactivation

**Figure 5:**
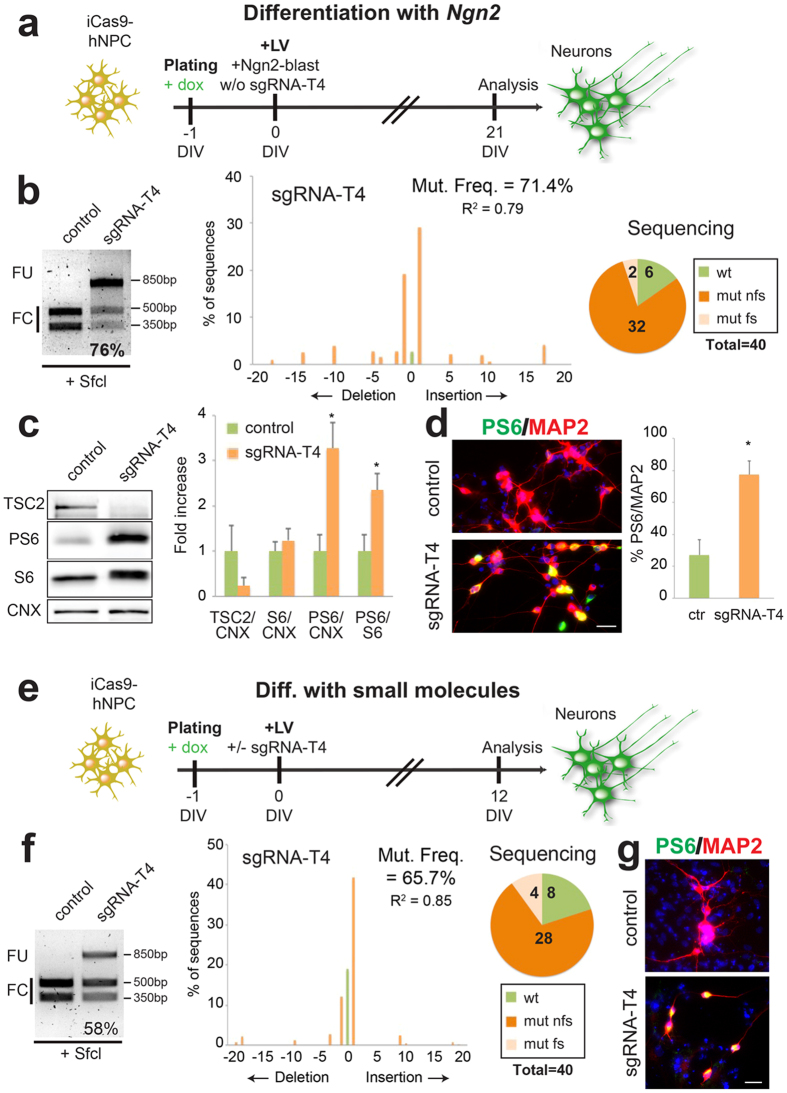
*TSC2* gene-inactivation in iCas9-hNPC-derived neurons leads to activation of the mTORC1 signaling. (**a**) Illustration of the experimental design to generate an enriched population of *TSC2*-targeted neurons from iCas9-hNPCs. The differentiation protocol, based on the forced expression of *Ngn2*, is coupled to *TSC2* gene targeting when the sgRNA-T4 guide is also transduced. Doxycycline (added at DIV-1) was maintained for all the experiment. (**b**) Left, SfcI RFLP assay to test indel mutation frequencies in control and sgRNA-T4-treated neurons. Middle, TIDE analysis carried out in the sgRNA-T4 cells. Right, number of wild type (wt), frameshift mutations (mut fs) and non frameshift mutations (mut nfs) found among 40 sequences analyzed in sgRNA-T4 sorted cells. (**c**) Western-blots to detect TSC2, PS6, S6 and Calnexin (CNX) protein levels in control and mutated neurons. Densitometric quantification of TSC2, PS6 and S6 relative to CNX or S6 protein levels in arbitrary units (n = 3 for TSC2, n = 4 for the others, *p < 0.05). (**d**) Double PS6/MAP2 immunostaining in the neuronal population. Quantification of PS6/MAP2 double positive cells respect to the total MAP2 positive cell population (n = 3, *p < 0.05). (**e**) Illustration of the experimental design to generate CRISPR/Cas9 targeted TSC2 neurons from iCas9-hNPCs differentiation using small molecules and morphogens. Doxycycline (added at DIV-1) was maintained for all the experiment. (**f**) RFLP, TIDE assays and number of mutated/40 total sequences analyzed in sgRNA-T4-treated neurons. (**g**) Immunostaining for PS6 and MAP2. Percentage of PS6/MAP2 positive cells relative to MAP2 positive cells. Hoechst was used to stain nuclei. Scale bar: 20 μm. Full-length gels and blots are included in Figure S8a–c.

Since the use of hNPCs derived from iPSCs is common in the studies of neuronal pathologies we thought to adapt our approach of combined gene mutagenesis and accelerated neuronal differentiation starting from hNPCs. Initially, iCas9-hPSC-derived NPCs were transduced with Ngn2-blast lentiviral particles expressing or not (control) the sgRNA-T4 (Figure S3c and Fig. 5a). Transduced iCas9-hNPCs were then differentiated in a neurobasal medium supplemented with B27 and BDNF. 21 days after infection, RFLP analysis showed that only in the presence of the sgRNA-T4 a significant *TSC2* gene targeting rate (76%) could be obtained in the hNPC-derived neurons (Fig. 5b). Moreover, the high efficiency of the method was confirmed in 6 independent experiments indicating the robustness and the reproducibility of this protocol (69.5 ± 1.8%). Similar results (71.5 ± 1.0%, n = 3 and Fig. 5b) were found when transduced iCas9-hNPC-derived neurons were analyzed by TIDE. By sequencing the cloned DNA fragments from the bulk neuronal population, we found that 85% of the sequences were mutated, with almost 94% harboring out-of-frame indels (Fig. 5b). By immunoblotting, we observed an evident reduction of the total TSC2 protein and a concomitant increase in PS6 levels (Fig. 5c). Accordingly, more PS6/MAP2 double positive cells were scored in the sgRNA-T4 compared to control targeted cells (Fig. 5d).Finally, to corroborate its robustness, this method was exploited in combination with neuronal differentiation of iCas9-hNPCs based exclusively on morphogens and chemical compounds^44^,^45^. iCas9-hNPCs were infected with a LV-U6 lentivirus co-expressing the sgRNA-T4 or not (control), neuronal cells were analysed after 12 days *in vitro* (DIV) (Fig. 5e). Indel rate was about 60% as assessed by RFLP and TIDE assays while 80% of the sequences cloned from the targeted sequence amplified from the bulk population were mutated (Fig. 5f). We observed an increase of nearly 3-fold in the PS6/MAP2 double positive cells in the mutated cell population, confirming the TSC2 protein reduction and the consequent mTORC1 activation (Fig. 5g).

### Generation of *TSC2* targeted human neurons from direct reprogramming of fibroblasts

**Figure 6:**
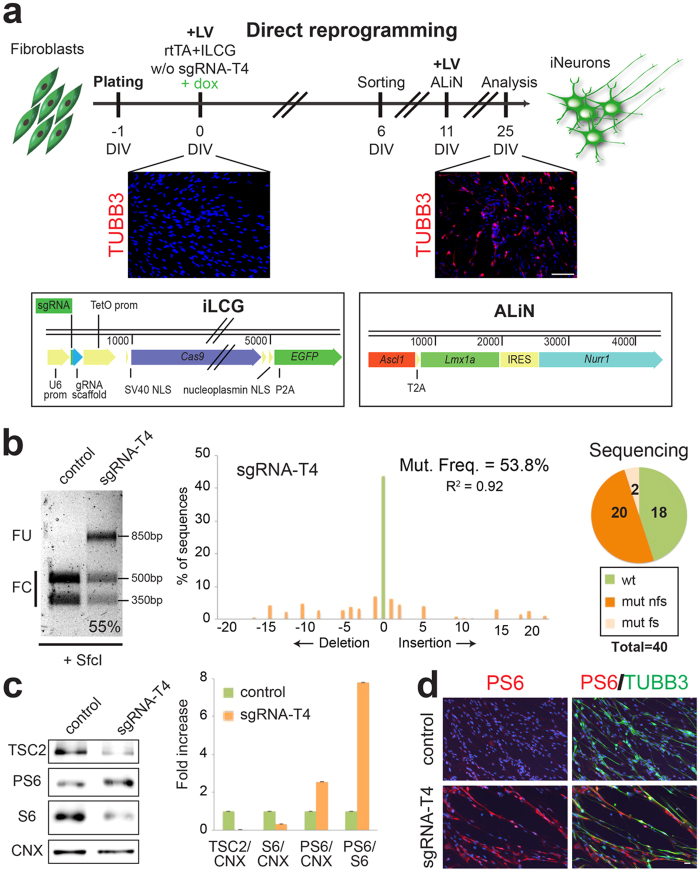
Straightforward *TSC2* gene inactivation in neurons directly reprogrammed from human fibroblasts. (**a**) Top, general strategy for the CRISPR/Cas9-mediated TSC2 gene inactivation during the direct neuronal conversion of fibroblasts. Doxycycline (added at DIV0) was maintained for all the experiment. Middle, TUBB3 immunostaining at 0 and 25 DIV showing the fibroblast conversion into neuronal cells. Bottom, design of the lentiviral vectors: iLCG (**a**1) expressing sgRNA-T4, Cas9 and *EGFP* under the TetO promoter and ALiN (**a**2) expressing *Ascl1*, *Lmx1a*, *Nurr1* linked by T2A and IRES sequences under the TetO promoter. (**b**) Left, RFLP analysis using SfcI enzyme in control and sgRNA-T4 induced neuronal cells. Middle, TIDE assay in the sgRNA-T4 induced neuronal cells. Right, number of wild type (wt), frameshift mutations (mut fs) and non frameshift mutations (mut nfs) found among 40 sequences analyzed in sgRNA-T4 induced neuronal cells. (**c**) Western-blots to detect TSC2, PS6, S6 and Calnexin (CNX) protein levels in control and mutated induced neuronal cells. Densitometric quantification of TSC2, PS6 and S6 relative to CNX or S6 protein levels in arbitrary units. (**d**) Immunostaining for TUBB3 and PS6. Hoechst was used to stain nuclei. Scale bar: 100 μm in (**a**) and 20 μm in (**d**). Full-length gel and blots are included in Figure S8d,e.

Finally, we asked whether this method could be applied for precise gene targeting in human neurons generated by direct reprogramming of fibroblasts. Clonal population of primary fibroblasts cannot be normally obtained and, therefore, this system can be a unique opportunity to introduce site- specific gene mutations during this process. For *TSC2* gene targeting, we generated a polycistronic lentiviral construct expressing the sgRNA-T4, together with a doxycycline-inducible SpCas9 and EGFP linked with a P2A self-cleavage sequence (Fig. 6a). Due to the significant size of the construct, causing the production of the virus with a relative low titer, we decided to FACS-sort the EGFP positive fibroblasts in order to enrich the relative amount of transduced cells. Subsequently, purified EGFP positive human fibroblasts were exposed to a second transduction step with a multicistronic lentivirus expressing the Ascl1, Lmx1a and Nurr1 genes, previously shown to be sufficient to promote the dopaminergic neuronal reprogramming of mouse and human fibroblasts^20^,^46^. 14 days after expression of the reprogramming genes, the resulting converted cell population was harvested and subjected to molecular assessment. As shown in Fig. 6b, about 55% indel rate was estimated by both RFLP and TIDE analyses. A similar frequency was subsequently confirmed by direct sequencing of the individual DNA molecules from the targeted genomic region (Figs 6b and S6). Despite the relative minor efficiency in *TSC2* gene targeting respect to hPSCs, total TSC2 protein was significantly reduced while PS6 levels were strongly increased in the mutated neuronal cells (Fig. 6c). Likewise, we scored 4-fold increase in PS6/TUBB3-double positive neuronal cells when the sgRNA-T4 was co-expressed with the other molecular components (Fig. 6d). Altogether, these results demonstrated that this method can be applied during direct neuronal cell reprogramming to obtain an efficient and precise targeting of a desired gene locus into the induced neuronal cells.

## Discussion

In this study we established a platform of fast and easily manageable tools that consent to investigate the role of a given gene during neuron formation. On this purpose we exploited the high flexibility and efficiency in gene editing of the CRISPR/Cas9 system, coupled with various approaches of neuronal differentiation. The intent was not only to reduce and simplify the efforts correlated with the generation of isogenic mutant cell lines, but also to complement this practice with a procedure that has a general applicability in many cell types and most genetic loci. The use of hPSCs is fundamental to model neuronal pathologies and study the genetic alteration that affect neuronal functionality, but it is also highly demanding in terms of conspicuous number of lines that need to be generated, maintained, and then differentiated into mature neurons for subsequent analysis^47^. We initially set this methodology using iCas9-hPSC lines that integrate the SpCas9 gene with an inducible promoter system^40^. In these cell lines, combined expression of the sgRNA and *Ngn2* by lentiviral transduction rapidly prompted a very mature neuronal phenotype^40^ and robust levels of gene inactivation in the bulk cell population with a simple procedure. In fact, for both *TSC2* and *KCNQ2* genes, we were able to reach 85% of gene editing frequency as verified by direct sequencing of the indels in the targeted domain.

To confirm that such frequencies of gene targeting in the bulk neuronal cultures were compatible with functional studies, we proved that cellular, molecular and functional alterations known to be direct consequence of *TSC2* and *KCNQ2* gene inactivation could be promptly identified and studied with this approach. Remarkably, we described molecular, functional and electrophysiological defects *in vitro* in neurons displaying concurrently a high degree of gene inactivation and neuronal maturation, thus proving strong evidence that this approach can be applied to study a wide array of neuronal alterations.

We then verified the validity of this system using wild-type hPSCs as well, confirming the possibility of generating site-specific mutations with high efficiency also in unmodified hPSCs. This method is a very convenient opportunity for studying the effects of inactivating genes of interest while limiting the production of isogenic hPSCs clones. Our intent was to streamline this process by introducing gene inactivating mutations concurrently with hPSCs neuronal induction, in order to complement the use of isogenic lines. Moreover in this set-up the introduction of indel mutations in the gene of interest is initiated during the very same process of neuronal differentiation rather than preceding it, excluding possible complications arising at the steps of neural progenitor specification or development.

It is also important to mention that in some instances gene inactivation can compromise hPSCs viability, proliferation or survival, resulting in the failure of clonal cultures^48^, whereas our approach would still be feasible in those cases with unaltered efficiency. Moreover, the same approach is convenient for other paradigms of neuronal differentiation such as NPCs and direct reprogramming of fibroblast. The derivation of stably growing NPCs is a common and manageable practice in hPSCs neuronal differentiation^43^,^44^. Since these cells are able to amplify but hardly grow in single cell, thereby limiting clonal selection, we reasoned that they represent an optimal target for this methodology. As a proof of concept, we derived iCas9-NPCs and subjected to CRISPR/Cas9 gene inactivation while promoting their differentiation using either *Ngn2* overexpression^41^ or a cocktail of small molecules associated to the selected sgRNA targeting *TSC2*^44^,^45^. In both cases the indel mutagenesis rate was very high (80–90%) and resulting to evident morphological and molecular alterations.

Given this remarkable level of indel mutations, it is likely that bi-allelic modification can occur within the targeted cell population, required to inactivate also recessive genes. This hypothesis is reinforced by the observation that in our settings, the occurrence of reading frame disruption (72%) significantly exceeds its mere statistical prediction, as also previously reported for other application of the CRISPR/Cas9 system^40^,^49^. In this perspective this procedure could be also indicated for multiple gene inactivation. Extending this system to Homologous Directed Repair (HDR), in order to introduce correct point mutations by means of single-stranded DNA Oligonucleotide (ssODN), is also intriguing, but depends on DNA transduction protocols that, in our cells, are yet very poor.

Direct neuronal reprogramming is a well-established procedure for the conversion of fibroblasts into functional neuronal cells. Forced expression of different transcription factor combinations can convert somatic cells into a wide array of neuronal phenotypes^20^,^21^,^22^,^23^,^24^,^25^,^26^. We focused on a direct cell reprogramming protocol previously devised by our laboratory^20^,^50^ and took advantage of the CRISPR/Cas9 system to introduce specific gene inactivating mutations during the process of neuronal conversion. The same *TSC2* targeting sgRNA employed in hPSCs and hNPCs studies was shown to be efficient for this purpose as well. Although this approach required the application of two different lentiviral vectors (the former carrying the reprogramming genes and the latter expressing the SpCas9 and the sgRNA), we obtained levels of *TSC2* gene inactivation sufficient for a reliable analysis of the morphological and biochemical alterations in the derived neuronal cells. These results have important applications in the field of direct cell reprogramming providing a system for rapidly testing the downstream effects associated to the inactivation of the gene of interest in the induced neuronal progeny. Moreover, this approach might be also valuable for studies aiming to unravel the molecular mechanisms controlling neuronal conversion downstream to the neuronal reprogramming factors.

Overall, we did not observe recurrent mutations at the off-target sites, as predicted by the bioinformatics tools, for both genes in the bulk targeted cell population. An unbiased analysis could be essential to confirm this specificity, however, it is likely that the impact of an off-target alteration in this context is less severe in comparison to a clonal cell line. In fact, a poorly represented genomic alteration in the bulk cell population can hardly lead to a consistent and discernible phenotypic effect.

Altogether, this methodological platform will strongly accelerate genetic studies in stem cell-derived and reprogrammed human neurons, providing a fast and simple procedure to induce precise genome modifications in hPSCs and fibroblasts and evaluate their functional consequences in the derived neuronal cells.

## Methods

### sgRNA design and selection

For sgRNA design, we selected a genomic region of ≈200 bp in the critical domain for protein function and affected by multiple disease causing mutations (Y1571X for TSC2 and Y284C for KCNQ2). We used CRISPR Design Tool (http://crispr.mit.edu) to search for sgRNA in this two regions of interest that are available for consultation at the following sites crispr.mit.edu/job/7103573089147924 and http://crispr.mit.edu/job/7052478635872826 respectively for human *KCNQ2* and human *TSC2*. Out of this we selected 6 sgRNAs with more than 50% of off-target score (see the Supplementary Table 1). sgRNAs were cloned in a LV-U6 vector as previously described^51^. To generate Cas9-expressing HEK293T cell line, we infected the cells with a polycistronic lentiviral construct that contains SpCas9 under the control of a constitutive promoter Ef1alpha. Infected cells were then selected with puromycin for 3 days. The expression of SpCas9 was assessed by qRT-PCR.

### Molecular cloning

sgRNAs were cloned in LV-U6 vector as previously described^51^. The LV-U6-filler plasmid was engineered replacing the TetO-NfiA cassette of the TetO-FUW- NfiA plasmid with the ‘U6-promoter-filler’ cassette PCR amplified and digested with SalI-XbaI from lentiCRISPR-v1 vector (Zhang’s lab, purchased from Addgene) restriction digested with XhoI-XbaI. iLCG-sgRNA vector were generated from the LV-U6-filler vector modified to introduce the TetO promoter (restriction digested XhoI-BamHI from Tet-O-FUW-NfiA vector and ligated in to LV-U6-filler plasmid restricted with the same enzymes) and the SpCas9-P2A-EGFP cassette (restriction digested and ligated BamHI-XbaI). This intermediate allowed for the generation of the iLCG construct by means of HpaI restriction fragment deletion and iLCG-SgRNA vectors by shuttling the U6-SgRNA cassette from the respective LV-U6 vector into the iLCG backbone using NotI-XhoI restriction enzymes. In order to replace the puromycin resistance of the pTet-O-*Ngn2*- puro construct (a kind gift of T.C. Sudhof) with the blasticidin resistance gene the entire cassette ‘TetO-*Ngn2*-T2A-puro’ was PCR amplified and cloned into a shuttle vector using TOPO TA cloning kit (Thermo Fisher). A T2A-Blast cassette was amplified to replace the T2A-Puro using AgeI XbaI/NheI ligation. Finally the TetO-*Ngn2*-T2A-Blast cassette was removed from the shuttle vector to be introduced into the LV-U6-sgRNA or LV empty vectors by means of BamHI-AgeI of restriction digestion and ligation to create respectively the Ngn2-blast and SgRNA-Ngn2-blast vectors. PCR amplified EGFP sequence was digested and introduced using AgeI-XbaI restriction enzymes, thus generating a TetO-*Ngn2*-P2A-EGFP-T2A-Puro vector. The restriction fragment NheI-SpeI containing the Ngn2 c-terminus, the P2A sequence and the EGFP cassette was then excided by NheI-SpeI to be inserted into the Ngn2-blast and Sg6-K-Ngn2-blast opened with NheI-XbaI digestion to generate NGB and sg6-K-NGB vectors. The TetO-ALiN vector was the product of multiple cloning steps. Initially, the murine Lmx1a sequence was PCR amplified in order to introduce and MCS and an in- frame T2A peptide sequence at the 5’end of its sequence, the PCR product was digested and cloned in the TetO-FUW-Ascl1 plasmid. Thus using the Hpa restriction site preceding the T2A sequence the murine Ascl1-sequence amplified was cloned into the vector. IRES and murine Nurr1 amplicons were subsequently inserted. Lentiviral particles were produced as previously described^52^.

### T7 Endonuclease I (T7EI), Restriction Fragment Length Polymorphism (RFLP) and Tracking of Indel DEcomposition (TIDE) assays

To extract genomic DNA, cells were incubated for 4–24 hours at 55 °C in a digestion buffer mix (100 mM Tris-HCl pH8, 200 mM NaCl, 5 mM EDTA, 0.5% SDS with proteinase K, Sigma). Then, genomic DNA was purified using isopropanol, washed with ethanol 70% and resuspended in water. The genomic regions that were flanking the sgRNAs target sites were amplified by PCR using 100 ng of genomic DNA as a template. For T7EI analysis, 200 ng of purified PCR products (Wizard SV gel and PCR Clean-Up System, Promega) were denatured and reannealed in NEB buffer 2 (New England Biolabs). Primers for TCS2 were (5′-3′): F, CTGCTGTGTGGCTCGAGGA, R, AGGCCGTACCTTGCATGA. Primers for KCNQ2 were: F, ATGGCTCGGTGGAGACCAC, R, TCCAGTCCTTGGGGAACA. We used the following protocol: 95 °C, 5 min; 95 °C–85 °C at −2 °C/s; 85 °C–25 °C at −0.1 °C/s; hold at 4 °C. Then, 10U of T7EI (New England Biolabs) was added and an incubation step at 37 °C for 15 min was performed. PCR products were analyzed on 2% agarose gels and imaged with a Gel Doc gel imaging system (Bio-Rad). The percentage of genome modification was obtained as previously described^53^. For RFLP assay, the digestion mix containing 500 ng of purified PCR products (Wizard SV gel and PCR Clean-Up System, Promega), 5U of SfcI enzyme in NEB buffer 4 (New England Biolabs) was incubated at 37 °C for 2 hours. Primers for TCS2 were: F, CTGCTGTGTGGCTCGAGGA, R, AAGACGGCTGAGGGTGAG. Next steps were performed as in T7EI assay, for quantification we estimated the intensity of the uncleaved fraction. For TIDE assay, purified PCR products were analyzed by Sanger sequencing (GATC Biotech). The quantification of genome modification was obtained using the TIDE software^54^. PCR amplified were also cloned using TOPO TA cloning kit (Thermo Fisher) and then, each clone was individually sequenced.

### Off-Target analysis

The potential off-target sites of both sgRNA-K6 and sgRNA-T4 were selected according to online tool: http://crispr.mit.edu. Complete off-target lists are available at the sites indicated in the “*sgRNA design and selection*” section, in the specific the sgRNA-K6 is #6 and the sgRNA-T4 is #18. In brief, we chosen the top 8 most likely off-targets hits for each sgRNAs falling in gene coding regions, as previously described in other works^7^,^41^,^55^. These sites were amplified by genomic PCR (primers are listed in Supplementary Table 2) and then underwent for both TIDE analysis and Sanger sequencing in order to assess the absence of alterations on bulk population. Then, the PCR products were cloned into a shuttle vector (TOPO TA cloning kit, Thermo Fisher) for direct sequencing of ten cloned amplicons.

### Cell cultures and differentiation

The iCas9-HUES9 line^39^ and a wild-type hiPSC cell line generated from neonatal fibroblasts obtained from ATCC were used throughout this study. Both were maintained in feeder-free conditions in mTeSR1 (Stem Cell Technologies) and seeded in HESC qualified matrigel (Corning)-coated 6-well plates. hNPCs were generated as previously described with small modifications^44^. Briefly, hPSCs were dissociated as cell clusters using Accutase (Sigma) and seeded onto low-adhesion plates in mTeSR1 supplemented with N2 (1:200), ThermoFisher Scientific), Pen/Strept (1%, Sigma), human Noggin (0.5 μg/ml, R&D System), SB431542 (5 μM, Sigma) and Y27632 (10 μM, Miltenyi Biotec). After 5 days, embryoid bodies were seeded onto matrigel-coated plates (1:100, matrigel growth factor reduced, Corning) in DMEM/F12 (Sigma) supplemented with N2 (1:100), non-essential amino acids (1%, MEM NEAA, ThermoFisher Scientific) and Pen/Strept. After 10 days, rosettes were dissociated with Accutase and plated onto matrigel coated-flasks in NPC media containing DMEM/F12, N2 (1:200), B27 (1:100, ThermoFisher Scientific), pen/strept (1%) and FGF2 (20 ng/ml, ThermoFisher Scientific). For the infection, hNPCs were dissociated with Accutase and plated on matrigel-coated 24-well plates (1 × 10^5^ cells per well) in NPC medium supplemented with doxycycline (2 μg/ml, Sigma). The day after, hNPCs were infected in NPC media overnight. Then, the medium was replaced by differentiation medium containing Neurobasal (ThermoFisher Scientific), glutamine (2 mM, Sigma), Pen/Strep (1%), B27 (1:50), human BDNF (10 ng/ml, PeproTech) and doxycycline (2 μg/ml). Doxycycline was maintained for all the experiment. To induce the iCas9-hNPC differentiation with small molecules, retinoic acid (1 μM, Sigma) and forskolin (5 mM, Sigma) were also added in the differentiation medium. Half of the medium was changed every 2–3 days. Differentiated cells were maintained for 12–21 days.

hPSC neuronal differentiation was performed as previously described^20^. After single cell dissociation with Accutase, hPSCs were plated on HESC qualified matrigel-coated 6-well plates (1,5 × 10^6^ cells per well) in mTeSR1 with Y27632 (10 μM) and doxycycline (2 μg/ml) in the case of iCas9-hPSCs. The day after, cells were incubated overnight with the virus. The medium was replaced by DMEM/F12 supplemented with N2 (1:100), non-essential amino acids (1%), penicillin/streptomycin (1%), BDNF (10 ng/ml), mouse laminin (0.2 μg/ml, Sigma) and doxycycline (2 μg/ml). Doxycycline was maintained for all the experiment. The day after, the cells were FACS-sorted and plated in the same medium supplemented with Y27632 (10 μM) on matrigel (1:30)-coated 24-well plates (2 × 10^5^ cells per well). Sorting gates were always pre-set using a matching GFP negative control (hPSCs cells transduced with Ngn2-blast). Negative control and GFP-expressing samples were generated in parallel and in the same conditions. For iCas9-hPSCs, the medium was replaced the day after by differentiation medium supplemented with Blasticidin (10 μg/ml, ThermoFisher Scientific) and Ara-C (5 μM, Sigma). For wild type hPSCs, differentiation medium was also supplemented with Puromycin (1 μg/ml) for 24 hours. Cells were analyzed when they were 7 days in culture.

For primary hippocampal neuronal cultures, brains from E18.5 mouse embryos were dissected in cold HBSS (Hank’s balanced salt solution, Gibco) supplemented with glucose 0.6% and 5 mM Hepes pH7.4 (Sigma). Embryonic hippocampi were then extracted, rinsed twice with HBSS and treated with trypsin 0.25% (Sigma) for 15 minutes at 37 °C. Afterwards, hippocampi were washed in HBSS, re-suspended in 5 ml of plating medium (Neurobasal medium (NBM), 10% FBS, 1% Pen/Strept antibiotics) and mechanically dissociated in single cells. Cells were seeded at a final density of 2 × 10^5 ^cells/MEA onto sterile MEAs pre-treated overnight with poly-L-lysine 2 mg/ml (Sigma), each inside a Petri dish filled with water to reduce evaporation. After 4 h of incubation at 37 °C the medium was completely replaced with 1 ml of cell culture medium (NBM, B-27, 1% pen/strept, 1 mM Glutamax and 10 mM Hepes pH7.4). Neurons were maintained for 19–22 days in a humidified 5% CO_2_ incubator at 37 °C. The 30% of the total amount of medium was changed every 2 days and 12 hours before the electrophysiological recordings.

For electrophysiology, iCas9-hPSCs were plated at 1 × 10^5^ cells per coverslip or 6 × 10^5^ cells per MEA. 48 hours after the infection, Blasticidin was added for 5 days and then, mouse glia isolated from P1/2 mouse cerebral cortex was added (5 × 10^4^ cells). At day 10 post-infection, FBS (2.5%, Sigma) was included in the medium. Cells were analyzed after at least 5 weeks. Doxycycline (2 μg/ml) was maintained for all the experiment. For the direct reprogramming we used human lung fibroblasts MRC-5 (ATCC) maintained in MEF medium (Dulbecco’s modified Eagle medium (DMEM; Sigma) containing 10% FBS (Sigma), non-essential amino acids (Life Technologies), sodium pyruvate and Pen/Strept. MRC-5 were infected with iLCG in MEF media and 16–20 h later, the medium was replaced with fresh MEF media containing doxycycline (2 μg/ml; Sigma). After 6 days, EGFP-positive cells were FACS-sorted and then, they were infected with ALiN lentivirus. After 48 h, the medium was replaced with neuronal inducing media (DMEM/F12 (Sigma), 25 μg/ml insulin (Sigma), 50 μm/ml transferrin (Sigma), 30 nM sodium selenite, 20 nM progesterone (Sigma), 100 nM putrescine (Sigma) and Pen/Strept (Sigma) containing doxycycline. Doxycycline was maintained for all the experiment. The medium was changed every 2–3 days and cells were analyzed after 25 days.

### Immunofluorescence

Cells were seeded on matrigel-coated glass coverslips and they were fixed for 20 min in ice in 4% paraformaldehyde (PFA, Sigma), solution in phosphate-buffered saline (PBS, Euroclone). Then, cells were permeabilized for 30 min in blocking solution, containing 0.2% Triton X-100 (Sigma) and 10% donkey serum (Sigma), and incubated overnight at 4 °C with the primary antibodies in blocking solution. The following antibodies were used: PS6 (1:200, Cell Signaling, Cat. 2211), MAP2 (1:500, Immunological Sciences, MAB-10334) and TUBB3 (1:500, Covance, MMS-425P). Then, cells were washed with PBS and incubated for 1 h at room temperature with Hoechst and with anti-rabbit or anti-mouse secondary antibodies conjugated with Alexa Fluor-488, Alexa Fluor-594, Alexa Fluor-647 (1:1000, ThermoFisher Scientific) in blocking solution. After PBS washes, cells were washed again and mounted.

### RT-PCR analysis

RNA was extracted using Trizol reagent (Sigma-Aldrich) and then, retrotranscribed using iScript Super Mix (Biorad). In quantitative real time PCR, Titan HotTaq EvaGreen qPCR mix (BioAtlas) was used and expression levels were normalized respect to *β-ACTIN* expression. Primers for human *KCNQ2* were (5′-3′): F, TGCTGTCCCGAATTAAGAGC, R, GACCTGCTTCTCCACCTTCC. Primers for human *β-ACTIN* were: F, ACCCCAGCCATGTACGTT, R, GGTGAGGATCTTCATGAGGTAG.

### Western-blot

Protein extracts were prepared in RIPA buffer (10 mM Tris-HCl pH7.4, 150 mM NaCl, 1 mM EGTA, 0.5% Triton and complete 1% protease inhibitor mixture, Roche Diagnostics). Protein was separated on SDS-polyacrylamide gel, transferred to a nitrocellulose membrane and probed with a primary antibody against: PS6 (1:1000), TSC2 (1:1000, Cell Signaling, D93F12 Cat. 4308), calnexin (1:5000; Sigma, Cat. C4731), S6 (1:1000, Cell Signaling), KCNQ2 (1:500, Abcam, ab22897), ACTIN (1:2000, Sigma, A3853) followed by horseradish-peroxidase-conjugated secondary antibody (1:5000, Dako). KCNQ2 and ACTIN were used in reducing but not in denaturing conditions. Finally, it was visualized using ECL chemiluminescence (GE Healthcare).

### Electrophysiological recordings

Individual slides containing cultured neurons derived from iCas9-hPSCs were transferred in a recording chamber mounted on the stage of an upright BX51WI microscope (Olympus, Japan) equipped with an optical filter set for EGFP detection (Semrock, Rochester, NY, USA). Cells were perfused with artificial cerebrospinal fluid (ACSF) containing (in mM): 125 NaCl, 3.5 KCl, 1.25 NaH_2_PO_4_, 2 CaCl_2_, 25 NaHCO_3_, 1 MgCl_2_, and 11 D-glucose, saturated with 95% O_2_ 5% CO_2_ (pH 7.3). The ACSF was continuously flowing at a rate of 2–3 ml/min at room temperature. Whole-cell patch-clamp recordings were performed on EGFP-expressing fluorescent cells using glass pipettes filled with a solution containing the following (in mM): 10 NaCl, 124 KH_2_PO_4_, 10 HEPES, 0.5 EGTA, 2 MgCl_2_, 2 Na_2_-ATP, 0.02 Na-GTP, (pH 7.2, adjusted with KOH; tip resistance: 4–6 MΩ). All recordings were performed using a MultiClamp 700B amplifier interfaced with a PC through a Digidata 1440 A (Molecular Devices, Sunnyvale, CA, USA). Data were acquired using pClamp10 software (Molecular Devices) and analyzed with GraphPad Prism 5 (LaJolla, CA, USA) and SigmaStat 3.5 (Systat Software Inc., San Jose, CA, USA). Voltage-clamp traces were sampled at a frequency of 10 kHz and low-pass filtered at 2 kHz. To isolate KCNQ-mediated currents, hyperpolarizing voltage steps (from −40 to −100 mV in 10-mV steps, 1 s) were induced (each step evoked 25 s after the previous one) and the resulting currents were recorded. The protocol was repeated in control conditions until the current steady-state amplitudes became stable. Subsequently, the M-current selective blocker XE991 (Sigma) was bath applied and the protocol repeated several times for 15–20 min. M-currents were obtained offline by electronically subtracting traces recorded in XE991 from control traces. The steady-state value of the subtracted currents was measured by averaging amplitudes over a 200-ms window near the step command offset and subsequently plotted against voltage.

### Microelectrode Array (MEA) experiments and analysis

Standard 60-electrode MEA chips with 30 μm electrode diameter, 200 μm inter-electrode spacing and an integrated reference electrode (Multichannel Systems GmbH, MCS GmbH, Reutlingen, Germany) were employed for the electrophysiological extracellular recording of both hPSC-derived neurons and primary hippocampal neurons activity after 5–6 weeks and 19–22 days *in vitro* (DIV) respectively. The equipment for MEA recordings consisted of a pre-amplifier stage (MEA-1060- Inv-BC-Standard, gain: 55, bandwidth: 0.02 Hz–8.5 kHz, MCS GmbH), an amplification and filtering stage (FA64S, gain: 20, bandwidth: 10 Hz–3 kHz, MCS GmbH) and a data acquisition device (USB-ME64, sampling frequency: 25 kHz, MCS GmbH). To keep the cultures at physiological temperature and limit pH and osmolarity upward drifts, MEAs were kept at 37 °C with a temperature-controlled plate below the MEA (TCO2, MCS GmbH) and were covered with gas selectively permeable membranes (ALA-MEAMEM, MCS GmbH).

Recordings were carried out under control condition and in presence of different concentrations of XE991. The spontaneous electrophysiological activity was monitored for 15 minutes at the beginning of each experiment, before drug administration, to reach a stable level of the electrical signal and then for 15 minutes for each XE991 concentration. Before administration, the drug was prepared by taking out of the chip 1/5 of the total volume of the medium, mixing it with a 5X dose of the compound and gradually returning it into the chip in order to limit mechanical alterations of the network. After each administration, a 1-minute wait was applied before starting the recording in order to permit drug diffusion and stabilize the activity. To analyze MEA data, an off-line spike detection was performed with MC_Rack Software (MCS GmbH) appointing a channel-specific threshold equal to 5-folds the standard deviation of the average noise amplitude during the first 500 ms of recording. The subsequent spike analysis was implemented in Matlab (The Mathworks, Natick, USA), as previously described^42^. In brief, MEA channels were considered active when they displayed a spike rate higher than 0.03 Hz. Network bursts (NB), i.e. recurrent events of synchronized firing encompassing simultaneously in different electrodes, were detected when the product of the number of active channels and the number of spikes considered in 25 ms bins was higher than 9 and when the minimum inter-NB interval was 100 ms, as previously described^42^. The considered parameters included the number of active channels, the average spike number on the active channels, the average number of channels involved in NB and the NB frequency. To obtain a dose-specific value for each parameter, values were averaged over the last 10 minutes of each XE991 concentration and data normalized over the last 10 minutes of the starting control condition.

### Quantifications and statistical analysis

All data are represented as the mean calculated between different cultures and the variation between cultures is depicted as the standard error of the mean (SEM). For each experiment, “n” indicates the number of independent cultures used. Analyses of significant differences between means were performed using two-tailed Student’s t tests. Statistical significance was set at: *p < 0.05. For MEA data, a statistical test for multiple dependent variables was performed (Friedman test + Wilcoxon test as post hoc assessment). Comparison of parameters values related to different culture types were performed by means of a statistical test for multiple independent variables (Kruskal-Wallis test + Mann-Whitney as post hoc assessment).

## Additional Information

**How to cite this article**: Rubio, A. *et al*. Rapid and efficient CRISPR/Cas9 gene inactivation in human neurons during human pluripotent stem cell differentiation and direct reprogramming. *Sci. Rep*. **6**, 37540; doi: 10.1038/srep37540 (2016).

**Publisher’s note:** Springer Nature remains neutral with regard to jurisdictional claims in published maps and institutional affiliations.

## Supplementary Material

Supplementary Information
