# Supplementary material for: Rapid and efficient CRISPR/Cas9 gene inactivation in human neurons during human pluripotent stem cell differentiation and direct reprogramming
Source: Sci Rep. 2016 Nov 18;6:37540. doi: 10.1038/srep37540 (PMC5114606; doi:10.1038/srep37540)
Supplement: Supplementary Information [file srep37540-s1.pdf]

Supplementary information

Rapid and efficient CRISPR/Cas9 gene inactivation in human neurons during human pluripotent stem cell differentiation and direct reprogramming

Alicia Rubio[1,*], Mirko Luoni[1,*], Serena G. Giannelli[1,*], Isabella Radice[2], Angelo Iannielli[1], Cinzia Cancellieri[1], Claudia Di Berardino[1], Giulia Regalia[2,3], Giovanna Lazzari[4], Andrea Menegon[2], Stefano Taverna[5], Vania Broccoli[1,6]

Figure S1

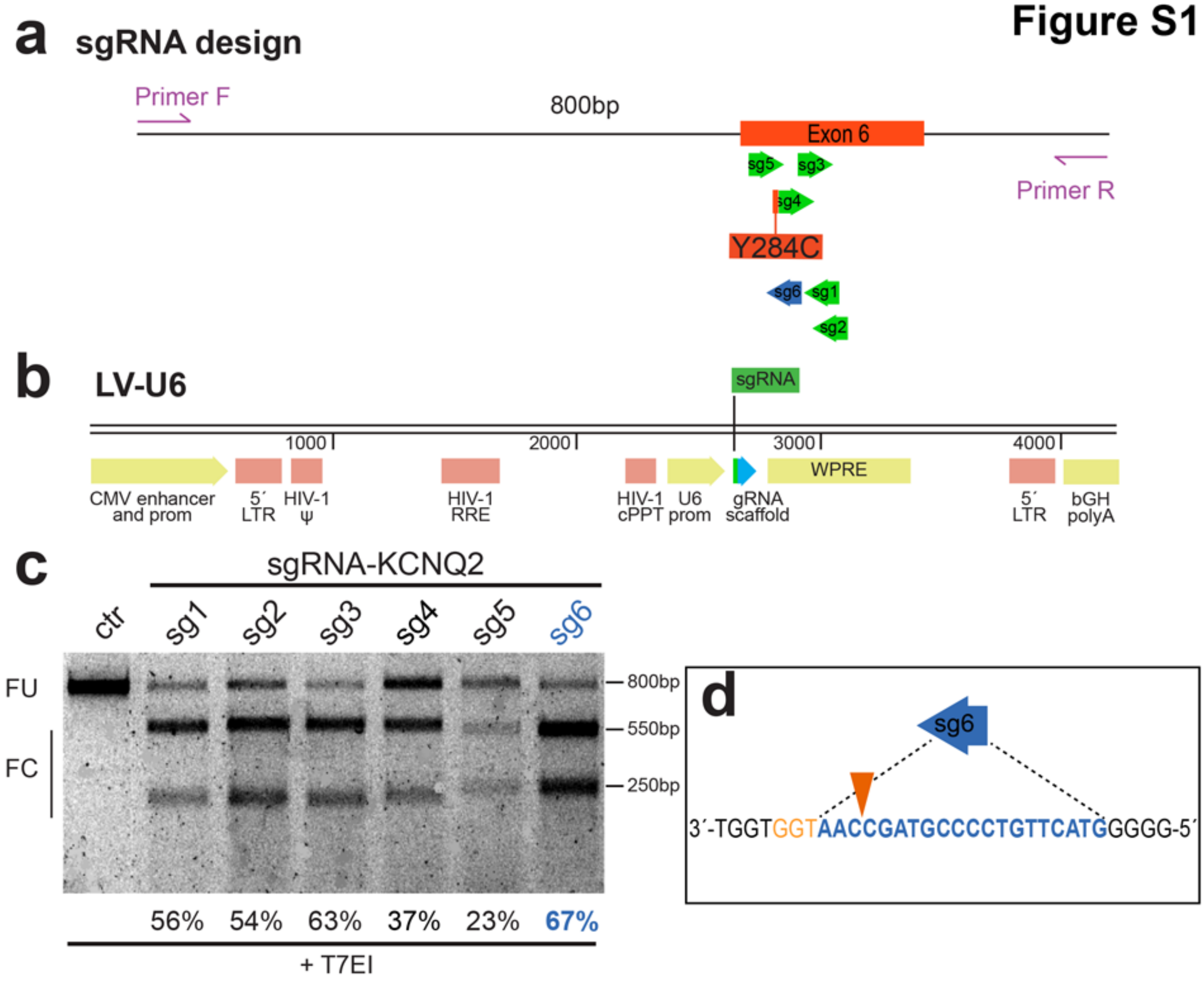

e

Gene	Chr	Sequence<br>3'-GTACTTGTCCCCGTAGCCAATGG-5'	Mismatches	Score	Chromatogramme
KCNV2	9	3'-GTACATGTCTCCGTAGCCCACGG-5'	3/4	0,91	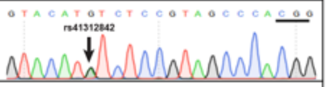
DNAH10	12	3'-GTACTCGTAGCCGTAGCCAAAGG-5'	3	0,83	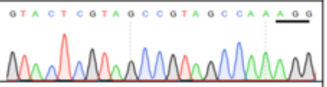
KCNB2	8	3'-GTAAATGTCACCATAGCCAACAG-5'	4	0,50	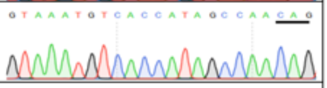
KCNF1	2	3'-GTAGATGTCGCCGTAGCCGACGG-5'	4	0,46	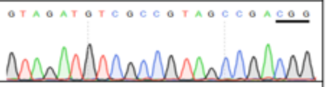
C3P1	19	3'-GTCCTGGACCCCGTAGCCCACAG-5'	4	0,30	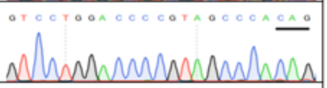
PGM1	1	5'-GTATTTGTCTCCTTAGCCATCAG-3'	4	0,24	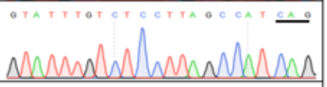
KCNA10	1	3'-GCACATGTCCCCATAGCCTACAG-5'	4	0,20	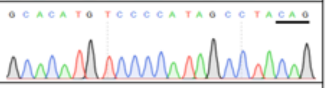
KCNC1	11	3'-GTACATGTCTCCATAGCCCAGGG-5'	4	0,017	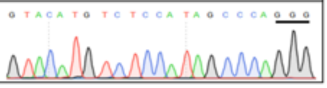

Figure S2

Sequencing *KCNQ2* in neurons derived from hPSCs

```
WT agagcctgcggtcccacagatcacgctgaccaccattggctacggggacaagtacccccagacctggaacggcaggc
1  agagcctgcggtcccacagatcacgctgaccaccattg-ctacggggacaagtacccccagacctggaacggcaggc -1nt
2  agagcctgcggtcccacagatcacgctgaccaccattgg-t-cagggacaagtacccccagacctggaacggcaggc -2nt
3  agagcctgcggtcccacagatcacgctgaccacca---gctacggggacaagtacccccagacctggaacggcaggc -3nt
4  agagcctgcggtcccacagatcacgctgaccaccattgg-----gggacaagtacccccagacctggaacggcaggc -5nt
5  agagcctgcggtcccacagatcacgctgaccaccattgg------ggacaagtacccccagacctggaacggcaggc -6nt
6  agagcctgcggtcccacagatcacgctgaccaccattgg------ggacaagtacccc-agacctggaacggcaggc -6nt;-1nt
7  agagcctgcggtcccacagatcacgctgaccacca-------cggggacaagtacccccagacctggaacggcaggc -7nt
8  agagc-----------------------------------tagggggacaagtacccccagacctggaacggcaggc -35nt
9  agagcctgcggtcccacagatcacgctgaccaccattggctacggggacaagtacccccagacctggaacggcaggc +1nt
                                          a
10 agagcctgcggtcccacagatcacgctgaccaccattggctacggggacaagtacccccagacctggaacggcaggc +1nt
                                          g
11 agagcctgcggtcccacagatcacgctgaccaccattgggtacggggacaagtacccccagacctggaacggcaggc +2nt
                                        tg
12 agagcctgcggtcccacagatcacgctgaccaccattggctacggggacaagtacccccagacctggaacggcaggc +4nt
                                        tcag
13 agagcctgcggtcccacagatcacgctgaccaccattggctacggggacaagtacccccagacctggaacggcaggc +9nt
                                      tccccgtag
14 agagcctgcggtcccacagatcacgctgaccaccattgg-------gacaagtacccccagacctggaacggcaggc +22nt;-7nt
                   agaatggtcccacagatcacgc
```

Figure S3

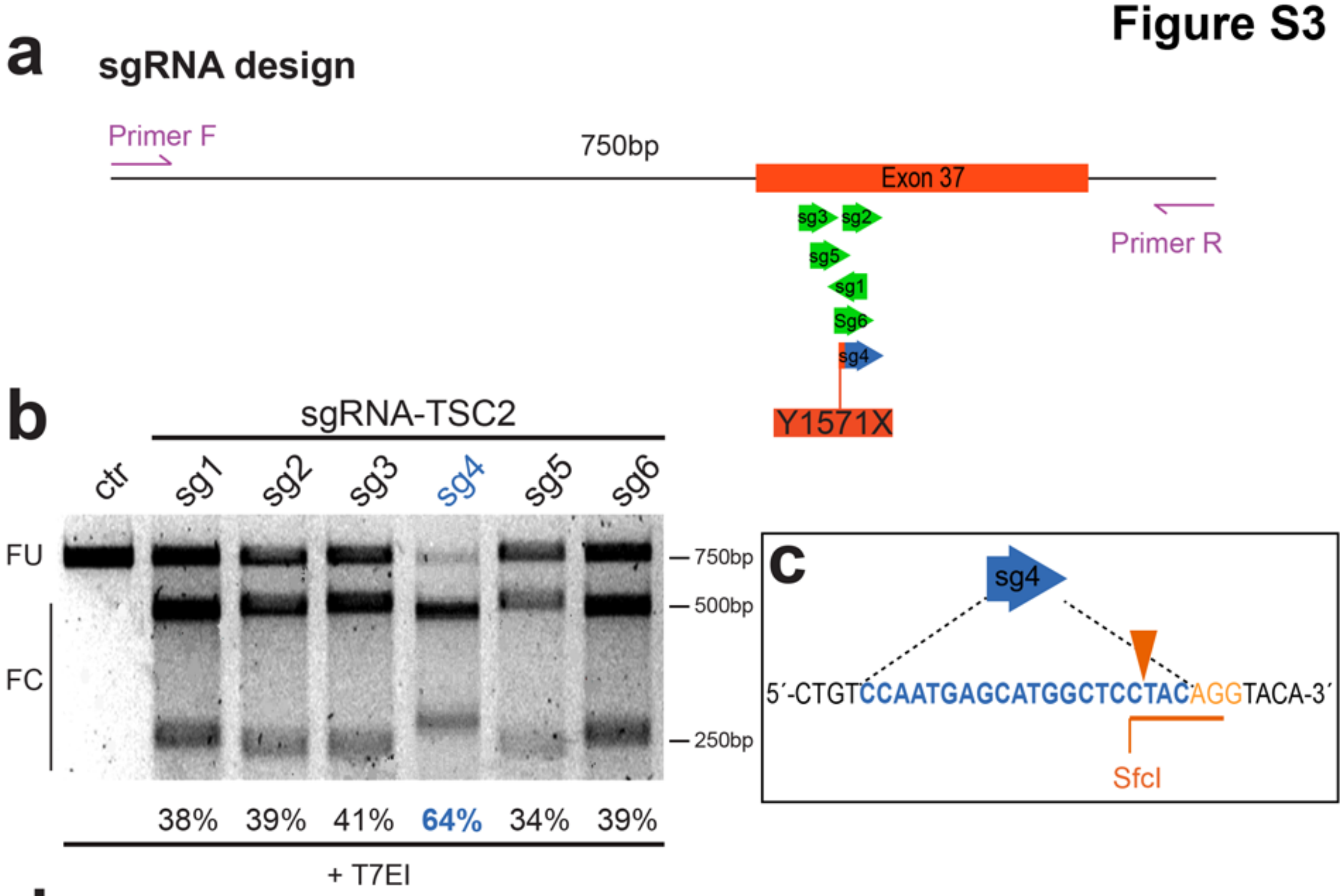

d

Gene	Chr	Sequence<br>5'-CCAATGAGCATGGCTCCTACAGG-3'	Mismatches	Score	Chromatogramme
TSSK4	14	5'-CCATTGGCCATGGCTCCTATGGG -3'	4	0,46	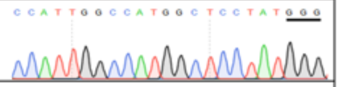
ENO1	1	3'-CCACAGAGCAGGGCTCCTATGAG-5'	4	0,37	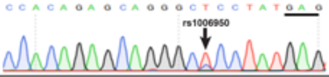
CCT8L2	22	5'-CCCAGCAGCATGGCTCCTCCGGG-3'	4	0,30	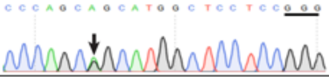
KIAA2012	2	5'-ATAATTAGCATGGATCCTACCGG-3'	4	0,14	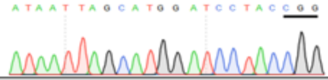
RPH3A	12	5'-CCCATGAGCATGGCTCATCTGAG-3'	4	0,082	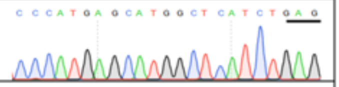
BDNF	11	5'-CCATTGAGCAAGGCACCTTCAAG-3'	4	0,074	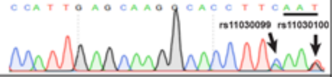
HFE2	11	3'-CCAAGGAGCTTGGCCCTCTACTGG-5'	4	0,063	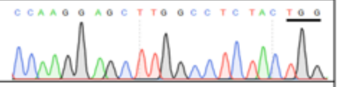
PABPC4	1	5'-CCTATGAGCATTGTTCCTATGAG-3'	4	0,049	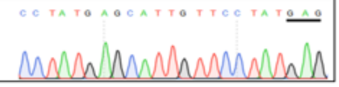

Figure S4

Sequencing *TSC2* in neurons derived from hPSCs

```
WT cagagcaacagcgagctcgccatcctgtccaatgagcatggctcctacaggtacacggagttcctgacgggcctggg
1  cagagcaacagcgagctcgccatcctgtccaatgagcatggctc-tacaggtacacggagttcctgacgggcctggg -1nt
2  cagagcaacagcgagctcgccatcctgtccaatgagcatggctc--acaggtacacggagttcctgacgggcctggg -2nt
3  cagagcaacagcgagctcgccatcctgtccaatgagcatggctcc-----gtacacggagttcctgacgggcctggg -5nt
4  cagagcaacagcgagctcgccatcctgtccaatgagcat-------acaggtacacggagttcctgacgggcctggg -7nt
5  cagagcaacagcgagctcgccatcctgtccaatgagcatggctc----------acggagttcctgacgggcctggg -10nt
6  cagagcaacagcgagctcgccatcctgtccaatgagcatgg----------tacacggagttcctgacgggcctggg -10nt
7  cagagcaacagcgagctcgccatcctgtccaatgag-------------ggtacacggagttcctgacgggcctggg -13nt
8  cagagcaacagcgagctcgccatcctgtccaa-----------------ggtacacggagttcctgacgggcctggg -17nt
9  cagagcaacagcgagctcgccatcctgtccaat------------------tacacggagttcctgacgggcctggg -18nt
10 cagagcaacagcgagctcgccatcctgtccaat-------------------acacggagttcctgacgggcctggg -19nt
11 cagagcaacagcgagctcgccatcctgtccaatgagcatggct-ctacaggtacacggagttcctgacgggcctggg -1nt; +1nt
                                              c
12 cagagcaacagcgagctcgccatcctgtccaatgagcatggctcctacaggtacacggagttcctgacgggcctggg +1nt
                                              c
13 cagagcaacagcgagctcgccatcctgtccaatgagcatggctcctacaggtacacggagttcctgacgggcctggg +2nt
                                               ct
14 cagagcaacagcgagctcgccatcctgtccaatgagcatggctcctacaggtacacggagttcctgacgggcctggg +4nt
                                               ctgt
15 cagagcaacagcgagctcgccatcctgtccaatgagcatggctcctacaggtacacggagttcctgacgggcctggg +12nt
                                              attcactccgtg
16 cagagcaacagcgagctcgccatcctgtccaatgagcatggctcctacaggtacacggagttcctgacgggcctggg +13nt
                                            attggagttcctg
```

Figure S5

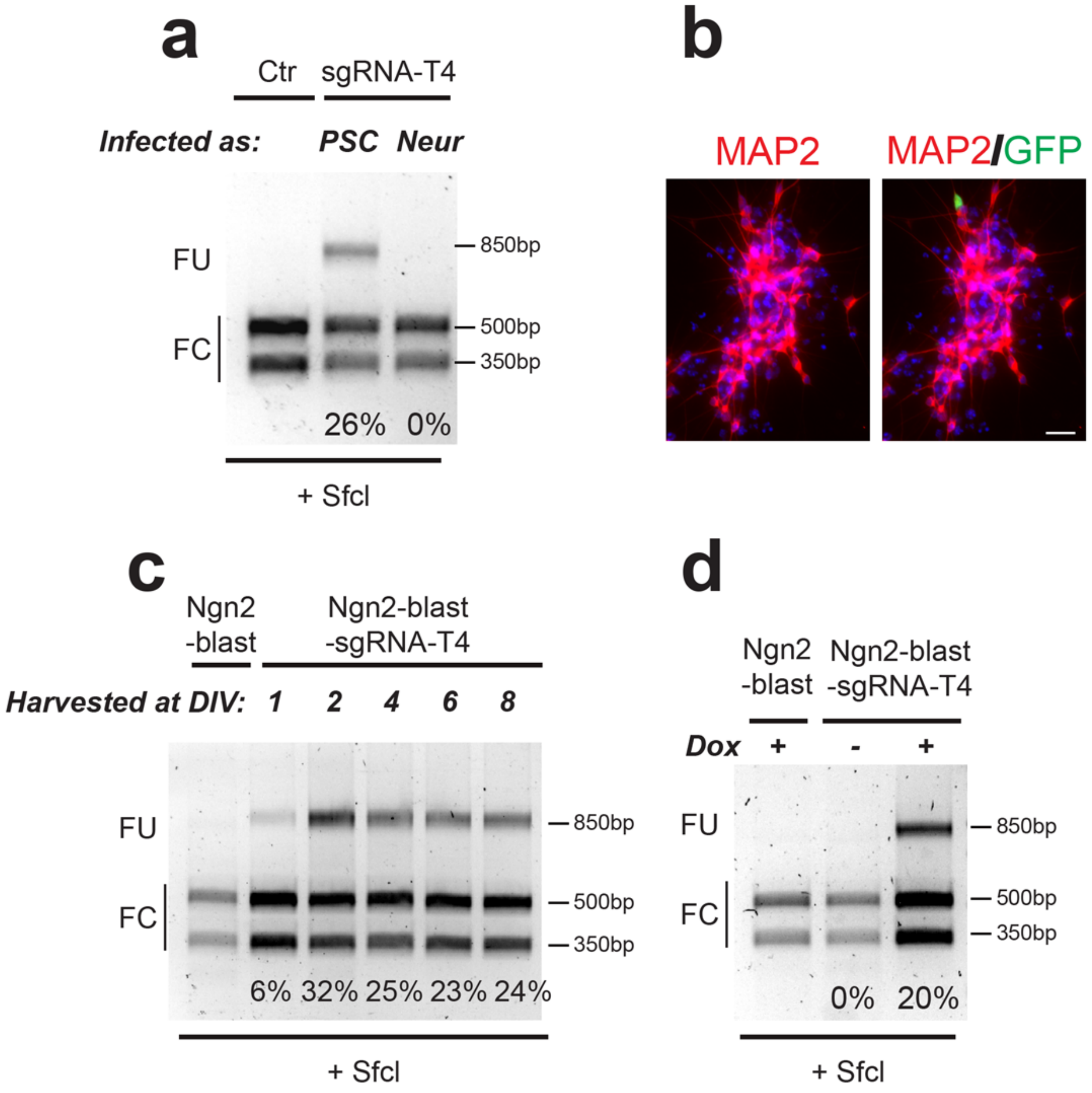

a
Ctr
sgRNA-T4
Infected as:
PSC
Neur
FU
FC
850bp
500bp
350bp
26%
0%
+ SfcI
b
MAP2
MAP2/GFP
c
Ngn2
-blast
Ngn2-blast
-sgRNA-T4
Harvested at DIV:
1
2
4
6
8
FU
FC
850bp
500bp
350bp
6%
32%
25%
23%
24%
+ SfcI
d
Ngn2
-blast
Ngn2-blast
-sgRNA-T4
Dox
+
-
+
FU
FC
850bp
500bp
350bp
0%
20%
+ SfcI

Figure S6

Sequencing *TSC2* in induced neurons derived from fibroblasts

```
WT cagagcaacagcgagctcgccatcctgtccaatgagcatggctcctacaggtacacggagttcctgacgggcctggg
1  cagagcaacagcgagctcgccatcctgtccaatgagcatggctcc-acaggtacacggagttcctgacgggcctggg -1nt
2  cagagcaacagcgagctcgccatcctgtccaatgagcatggct-----aggtacacggagttcctgacgggcctggg -5nt
3  cagagcaacagcgagctcgccatcctgtccaatgagcatggct---------at-tggagttcctgacgggcctggg -8nt;-1nt
4  cagagcaacagcgagctcgccatcctgtccaa------------------------------------gggcctggg -37nt
5  cagagcaacagcgagctcgcca--------------------------------acggagttcctgacgggcctggg -37nt
6  cag-----------------------------------------------gtacacggagttcctgacgggcctggg -47nt
7  cagagcaacagcgagctcgccatcctgtccaatgagcat-------acaggtacacggagttcctgacgggcctggg -7nt;+2nt
                                                   /\
                                                   gg
8  cagagcaacagcgagctcgccatcctgtccaatgagcatggctcctacaggtacacggagttcctgacgggcctggg +1nt
                                               /\
                                               c
9  cagagcaacagcgagctcgccatcctgtccaatgagcatggctcctacaggtacacggagttcctgacgggcctggg +2nt
                                                /\
                                                ct
10  cagagcaacagcgagctcgccatcctgtccaatgagcatggctcatacaggtacacggagttcctgacgggcctggg +4nt
                                                /\
                                                tggt
```

Figure S7

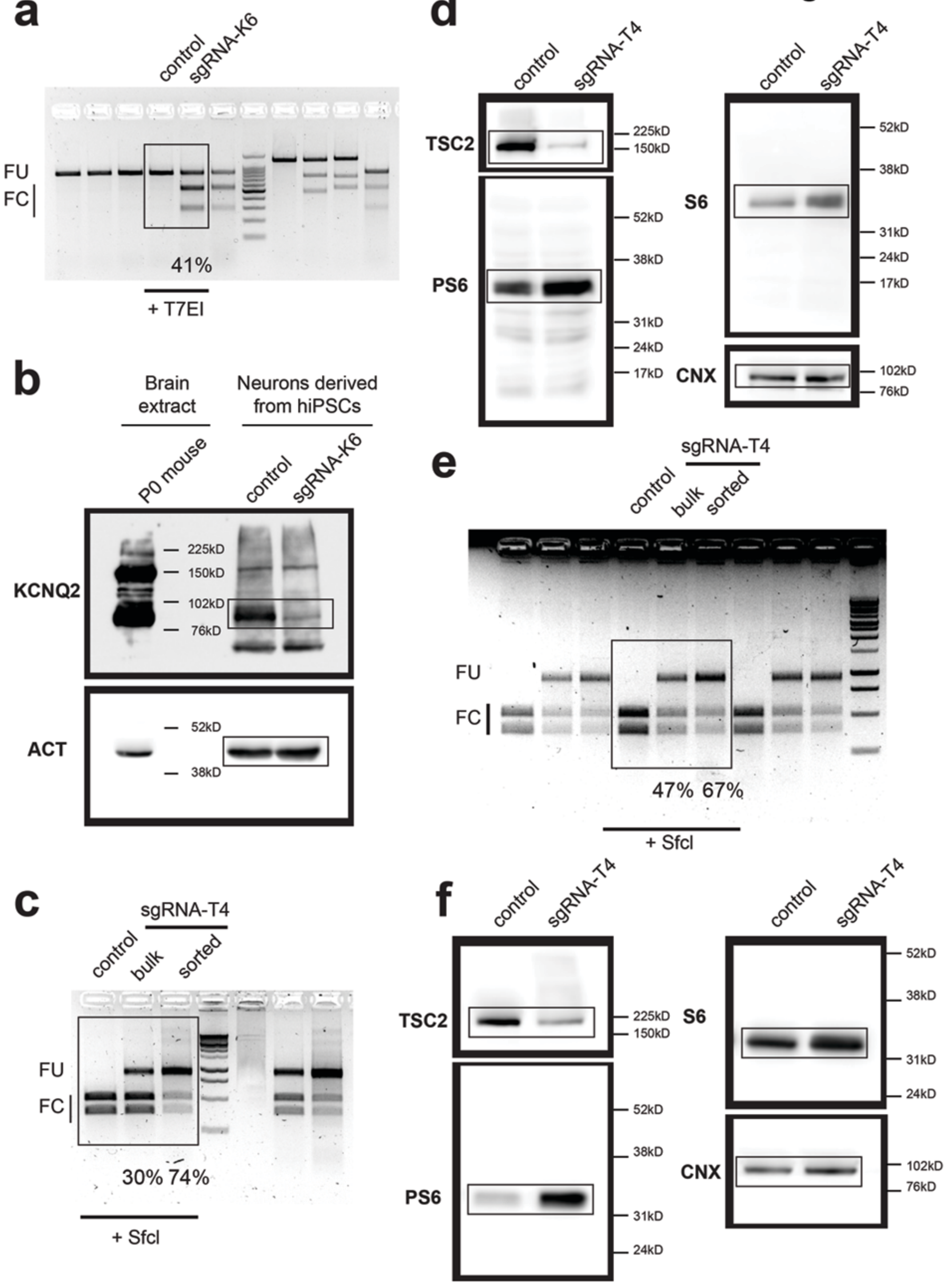
a
control
sgRNA-K6
FU
FC
41%
+ T7EI
b
Brain extract
Neurons derived from hiPSCs
P0 mouse
control
sgRNA-K6
KCNQ2
225kD
150kD
102kD
76kD
ACT
52kD
38kD
c
sgRNA-T4
control
bulk
sorted
FU
FC
30% 74%
+ SfcI
d
control
sgRNA-T4
TSC2
225kD
150kD
PS6
52kD
38kD
31kD
24kD
17kD
S6
CNX
102kD
76kD
e
sgRNA-T4
control
bulk
sorted
FU
FC
47% 67%
+ SfcI
f
control
sgRNA-T4
TSC2
225kD
150kD
PS6
52kD
38kD
31kD
24kD
S6
CNX
102kD
76kD

Figure S8

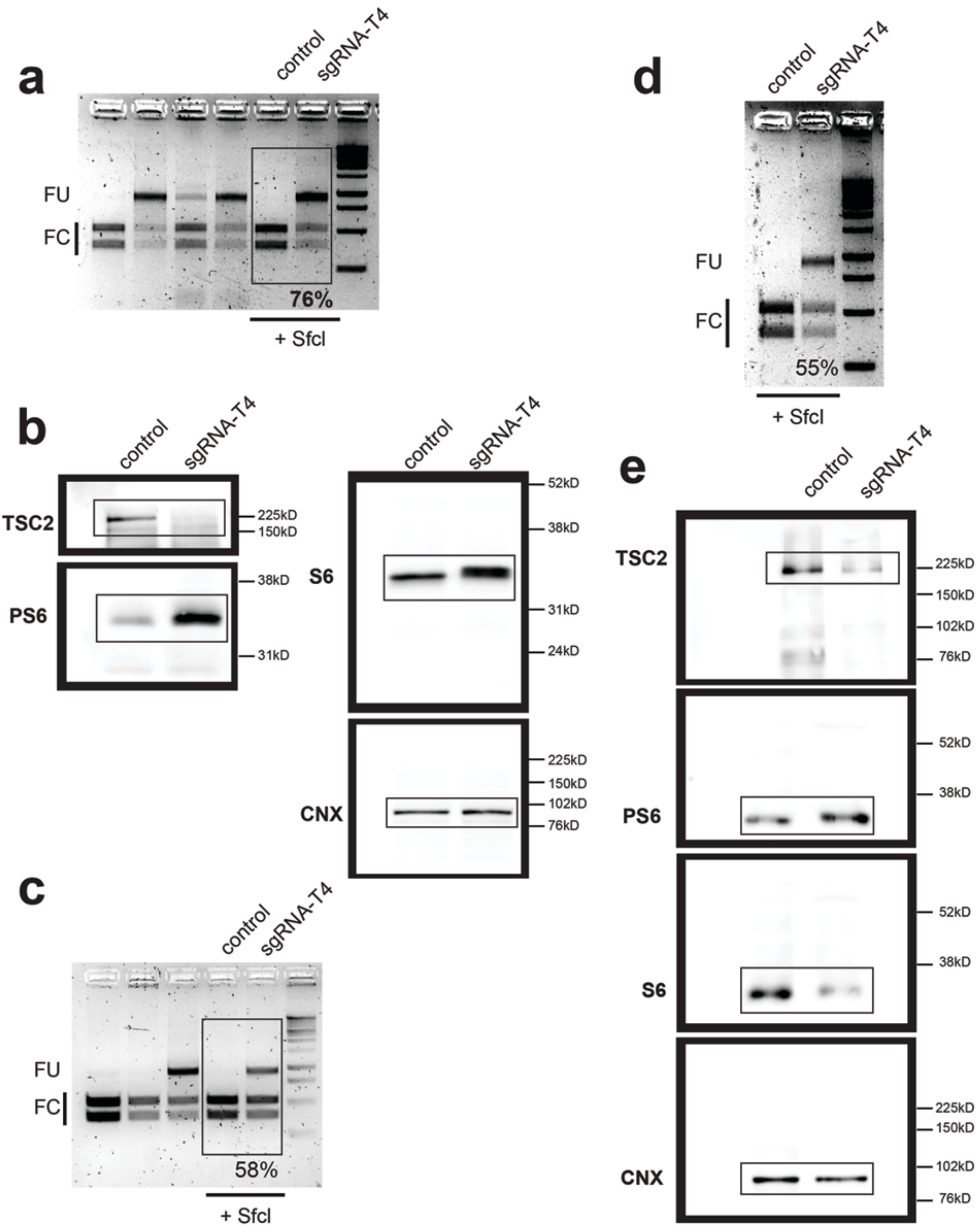
a
control
sgRNA-T4
FU
FC
76%
+ Sfcl
b
control
sgRNA-T4
TSC2
225kD
150kD
PS6
38kD
31kD
S6
52kD
38kD
31kD
24kD
CNX
225kD
150kD
102kD
76kD
c
control
sgRNA-T4
FU
FC
58%
+ Sfcl
d
control
sgRNA-T4
FU
FC
55%
+ Sfcl
e
control
sgRNA-T4
TSC2
225kD
150kD
102kD
76kD
PS6
52kD
38kD
S6
52kD
38kD
CNX
225kD
150kD
102kD
76kD

Supplementary information

Supplementary Figure 1 related to Figure 1: sgRNA design and validation for *KCNQ2* gene editing and analysis of predicted off-targets.

a. SgRNA design for targeting the *KCNQ2* gene. The selected region is surrounding the Y284C mutation on exon 6 encoding for the P-loop domain of the channel. **b.** Schematic view of the LV-U6 lentiviral vector structure. **c.** T7EI analysis of the DNA cleavage efficiency for each of the selected sgRNA in Cas9-expressing HEK293T. **d.** Sequence of the sgRNA-K6 guide and its PAM domain are indicated in blue and orange, respectively, orange arrow: expected site of double strand break. **e.** Analysis of the 8 most likely off-target genomic sites. In the sequences, nucleotide mismatches respect to the sgRNA sequence are highlighted in red and PAM domains are in orange. In the chromatogrammes, PAM sequences are underlined. Note the single nucleotide polymorphism (SNP) rs41312842 in *KCNV2*. Mismatches are indicated. Exclusion and inclusion of SNPs is also taken into account.

Supplementary Figure 2 related to Figure 1: CRISPR/Cas9 mutation spectrum in *KCNQ2.*

Representative sequences of sgRNA-K6 mutated neurons. Nucleotide substitutions, deletions and insertions are indicated in red. sgRNA and PAM sequences are shown in green and orange, respectively.

Supplementary Figure 3 related to Figure 3: sgRNA design and validation for CRISPR-mediated *TSC2* gene inactivation and analysis of the predicted off-target genomic sites.

a. Schematic illustration of the sgRNA design in the *TSC2* gene. The selected genomic region is centered on the Y1571X disease causing mutation in exon 37 coding for the GAP domain of the TSC2 protein. **b.** T7EI analysis of DNA cleavage efficiency for the selected sgRNAs in stable Cas9-expressing HEK293T. **c.** Sequence of the most efficient RNA guide (sgRNA-T4) and PAM domain are shown in blue and orange, respectively, orange arrow: expected site of double strand break. **d.** Analysis of the 8 most likely off-target genomic sites. In the sequences, nucleotide mismatches respect to the sgRNA sequence are highlighted in red and PAM domains are in orange. In the chromatogrammes, PAM sequences are underlined. Note the SNP rs1006950 in *ENO1*, the silent SNP in *CCT8L2*, rs11030099 and rs11030100 in *BDNF*. Mismatches are indicated. Exclusion and inclusion of SNPs is also taken into account.

Supplementary Figure 4 related to Figure 3: CRISPR/Cas9 mutation spectrum in *TSC2.*
Representative sequences of sgRNA-T4 mutated neurons. Nucleotide substitutions, deletions and insertions are indicated in red. sgRNA and PAM sequences are shown in green and orange, respectively. In yellow are highlighted the retained SfcI site.

Supplementary Figure 5 related to Figure 3: *TSC2* gene inactivation by CRISPR/Cas9 depending on the cell type infected and doxycycline treatment
a. SfcI RFLP analysis performed in iCas9-hPSC-derived neurons (at DIV10). The sgRNA-T4 was transduced either in non-differentiated hPSCs (at DIV0) or in post-mitotic post-mitotic neurons (at DIV7). **b.** Double immunostaining for GFP and MAP2 in post-mitotic neurons infected with a GFP expressing lentivirus. Nuclei were stained with Hoechst. Scale bar, 20 μm. **c.** SfcI RFLP assay in iCas9-hPSC infected with Ngn2-blast or Ngn2-blast-sgRNA-T4. Cells were harvested at different time points as indicated. **d.** SfcI RFLP assay in iCas9-hPSC-derived neurons (at DIV8). iCas9-hPSC were infected with Ngn2-blast or Ngn2-blast-sgRNA-T4. Doxycycline was not added in one of the samples transduced with the lentiviral vector Ngn2-blast-sgRNA-T4.

Supplementary Figure 6 related to Figure 6: CRISPR/Cas9 mutation spectrum in the *TSC2* gene in directly reprogrammed neurons.
Representative sequences of CRISPR mutated in directly reprogrammed neurons. Nucleotide substitutions, deletions and insertions are indicated in red. sgRNA and PAM sequences are shown in green and orange, respectively. In yellow are highlighted the retained SfcI site.

Supplementary Figure 7 related to Figure 1, 3 and 4: Full-length gels and western-blots.
a. Gel of Figure 1b. **b.** Blots of Figure 1d. As a positive control sample we used an enrichment of plasma membrane proteins obtained from brains of postnatal 0 (P0) mice. **c.** Gel of Figure 3b. **d.** Blot of Figure 3c. **e.** Gel of Figure 4b. **f.** Blots of Figure 4c. Cropped areas are indicated.

Supplementary Figure 8 related to Figures 5 and 6: Full-length gels and western-blots.
a. Gel of Figure 5b. **b.** Blots of Figure 5c. **c.** Gel of Figure 5f. **d.** Gel of Figure 6b. **e.** Blots of Figure 6c. Cropped areas are indicated.

Supplementary Table 1. List of the sgRNA guides used in this study

Supplementary Table 2. List of the primers used to amplify off-targets
